# Management‐Driven Effects in Soil Physicochemical Properties and Microbial Communities in Eastern Amazonian Agroforests

**DOI:** 10.1111/1758-2229.70432

**Published:** 2026-10-11

**Authors:** Rafael Barty Dextro, Maike Rossmann, Anderson Santos Freitas, Juan Andres Domini, Guilherme Lucio Martins, Uchida Shoichiro, Luiz Lehmann Coutinho, André Freire Cruz, Naoaki Ono, Siu Mui Tsai

**Affiliations:** ^1^ Centre for Nuclear Energy in Agriculture (CENA) University of São Paulo (USP) Piracicaba Brazil; ^2^ Superior School of Agronomy “Luiz de Queiroz” (ESALQ) University of São Paulo (USP) Piracicaba Brazil; ^3^ Data Sciences Center Nara Institute of Science and Technology (NAIST) Nara Japan; ^4^ Graduate School of Life and Environmental Sciences Kyoto Prefectural University (KPU) Kyoto Japan

**Keywords:** Amazon, land use, management practices, soil microbiome, sustainable agriculture

## Abstract

Agroforestry systems (AFS) are a promising bioeconomic strategy for sustainable land use and soil recovery in tropical regions. However, the potential effects of different management practices on soil microbial communities remain poorly understood. This study compared two neighbouring AFS in Tomé‐Açu (PA, Brazil) with similar land‐use, size, and productivity but contrasting management: organic inputs (AGF19) vs. chemical fertilization (AGF27), alongside a native forest reference area. Soil samples were evaluated through physicochemical properties, litter composition, and amplicon sequencing (16S rRNA and ITS) across three soil depths. Despite similar soil and litter characteristics, AGF19 showed a lower abundance of phytopathogenic fungi and associations with beneficial fungal taxa as biomarkers. In contrast, AGF27 exhibited signs of a microbiota shift towards a stress‐related community. These findings could be partially associated with modest management changes like favouring organic inputs, influencing microbial diversity, functional guilds, and soil chemistry. Differences in management could be potentially linked with the identification of a phytopathogenic fungal taxon (e.g., *Fusarium*) as a biological marker of the community sampled in the AGF27 soil. Although different from the native forest, the results strengthen the role of AFS as a sustainable agroecological strategy. Regular soil monitoring and microbiome‐informed practices are essential for sustainable management.

## Graphical abstract

Agroforestry management effects on soil microbiota were assessed in Eastern Amazon systems. Soil properties were similar, but microbiomes differed with management practices. Organic inputs reduced phytopathogenic fungi and promoted beneficial microbial taxa. Chemically managed systems showed signs of stress‐associated microbial shifts. Microbiome‐informed management can enhance sustainability in Amazonian agroforestry.

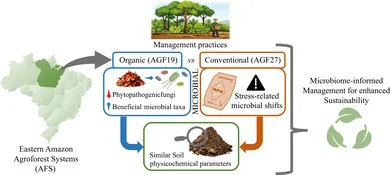

## 1 Introduction

Global food production faces a dual challenge: ensuring food security while conserving natural resources on a planet under increasing ecological and climatic pressure. In this context, agroforestry systems (AFS) have emerged as a practical and scalable alternative that balances agricultural productivity, biodiversity conservation, and soil biotic and abiotic conditions that favour plant development and the maintenance of a diverse microbial community (Dollinger and Jose 2018; Fahad et al. 2022). By mirroring forest structures through mixed plants cultivation, AFS offer a more sustainable alternative to monoculture and pasture (Sousa et al. 2024). This aligns with several key Sustainable Development Goals (SDGs) established by the United Nations, particularly SDG 2 (Zero Hunger), 12 (Responsible Consumption and Production), 13 (Climate Action), and 15 (Life on Land), by promoting sustainable and responsible agriculture, efficient land use, and counteraction against climate change grounded on nature‐based solutions (Goparaju et al. 2020).

Although the macroecological and socioeconomic benefits of AFS are well documented (Reyes et al. 2005; Jaramillo et al. 2013; Deltour et al. 2017; Liu et al. 2019), less attention has been given to agricultural management practices and how they might affect belowground networks and soil microbiota. This unseen soil component plays a critical role in nutrient cycling (Bhattacharyya and Furtak 2022), resilience to environmental stress, biological control of pathogens (Bhattacharjee and Dey 2014), and long‐term ecosystem stability via plant–soil feedback (Van der Putten et al. 2013; Saleem et al. 2019). This relationship results in enhanced plant growth and production yield (Gfeller et al. 2023), providing a direct benefit for the assessment and understanding of the soil's microbiome. As a key driver of ecosystem functionality, it is safe to assume that there is no sustainable agriculture without a healthy soil microbiome.

Despite the increasing recognition of agroforestry as a nature‐based solution for sustainable agriculture (Crowther et al. 2024), a major knowledge gap remains regarding how long‐term management practices within productive agroforestry systems shape belowground microbial communities and their ecological functions. Most studies have focused on the broad benefits of agroforestry compared with conventional agriculture or deforestation scenarios, while fewer have investigated whether conditions within established agroforests promote soil microbial assemblages comparable to those found in native ecosystems. The extent to which chemical fertilization and liming influence microbial diversity in Amazonian agroforestry landscapes remains poorly understood.

This study represents a comparison of individual case‐study sites with different management regimes to observe momentary differences in both chemical and microbial soil composition after almost two decades of agroforestry establishment, rather than replicated management treatments to fully describe their effects on soil properties. We sampled two adjacent AFS and a native forest reference in Tomé‐Açu (Pará, Eastern Amazon), which share a similar land‐use history but differ in their management practices. The 19‐years‐old AFS (named here AGF19) employs predominantly organic inputs, while the 27‐years‐old (AGF27) relies on chemical fertilizers and frequent liming. Our objective was to assess local differences on soil physicochemical characteristics and microbial communities of these managed‐agroforests and if they more closely align with those conditions found in preserved forests. This research takes an innovative approach by not only evaluating soil fertility and structure, but also examining how conditions on real‐life on‐going productive agroforestry system might have influenced the microbial assemblages responsible for sustaining critical ecosystem services. In addition to advancing fundamental knowledge in microbial ecology and soil science, the findings have direct applications in policy development, rural advance, and sustainable land management. They provide practical insights for farmers and policymakers seeking productive systems with reduced environmental degradation. By exploring the current soil conditions of productive agroforestry regimes, this work contributes to a better understanding of the sustainability in Amazonian agricultural landscapes.

## 2 Materials and Methods

### 2.1 Site Description and Experimental Design

This study was performed at two rural properties in Tomé‐Açu (PA, Brazil) with established agroforestry systems based on the mixed cultivation of several fruit‐producing plant species. Açai ( *Euterpe oleracea* ) and cacao ( *Theobroma cacao* ) are planted within square plots with other tree species (like banana *Musa acuminata* , cupuaçu *Theobroma grandiflorum* and Pará nut *Bertholletia excelsa* ) planted at random on the outside of the plots. Landowners gave their consent for soil sampling on their properties after being informed about the study's goals. AGF19 (2°28′11″ S, 48°18′16″ W) is a 19‐year‐old farm with 117 ha of productive area, and AGF27 (2°28′37″ S, 48°18′01″ W) is a 27‐year‐old farm with 90 ha reserved for plantation. Both possess similar overall fruit production (according to landowners). Used as a control, a native forest area within the same municipality (35 km away from both AFS) was also sampled (Figure 1A–C). Both farms, in neighbouring land and owned by brothers, have a very similar context of land use before the establishment of the AFS. After the removal of the native forest, both areas served as *Eucalyptus* plantations, with a short period of grassland followed by farming (horticulture, passionfruit, and black pepper). AGF19 management is less intensive, with the use of organic fertilizers in intervals of around 4 months, with no current soil pH treatment (over 2 years since last application of lime) and occasional weeding and pruning. AGF27 has a more direct and hands‐on management, with more frequent lime applications to reduce soil acidity, usage of chemical fertilizers in three to four rounds per year, and recurrent pruning. Specific information concerning dosages and products used was deemed private by farmers. Both sites use automatic irrigation systems and keep the naturally deposited aboveground litter composed of leaves, fruits and branches on the soils' surface.

**FIGURE 1:**
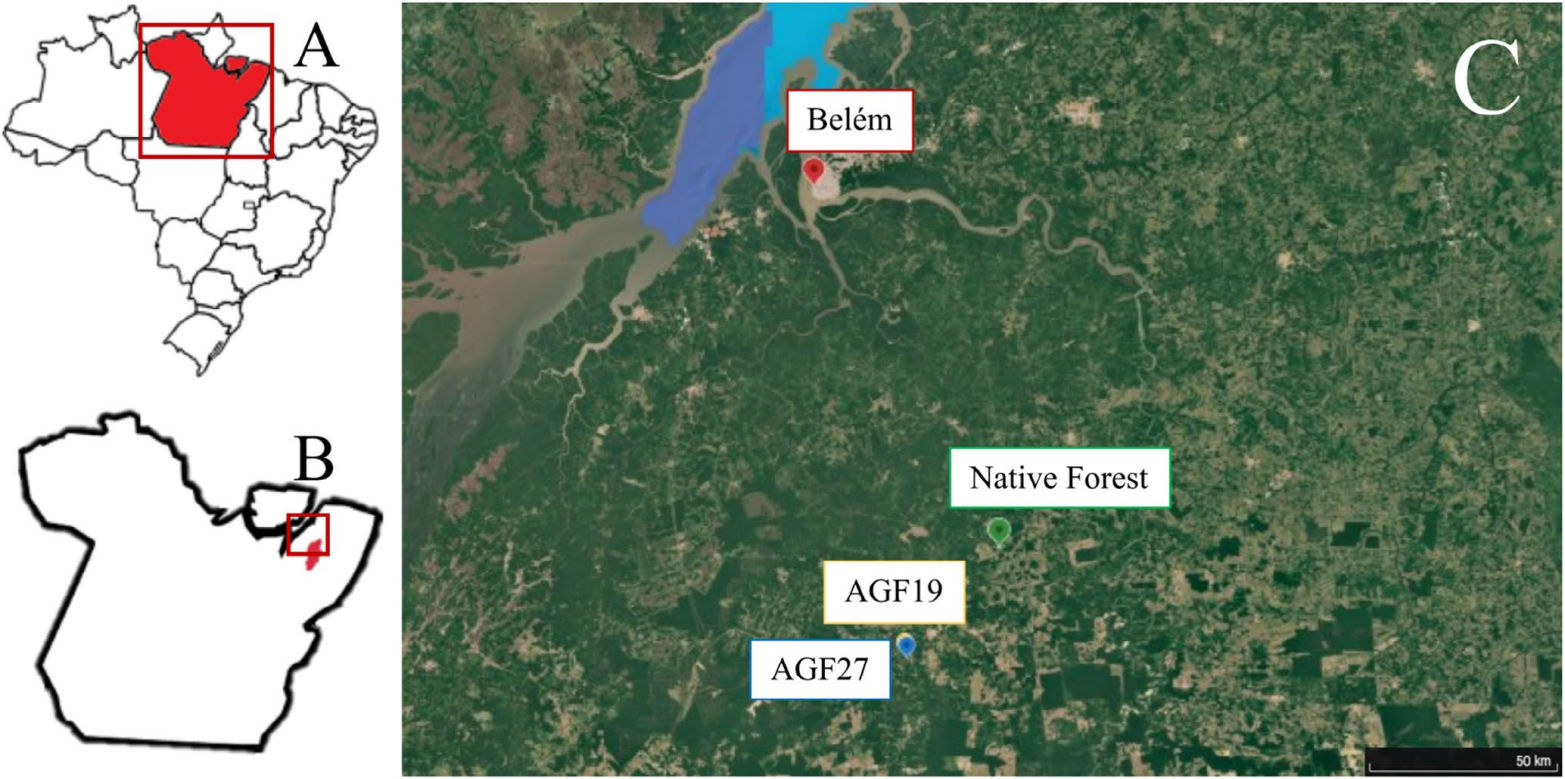
(A) Map of Brazil highlighting the Pará state (in red), (B) map of Pará state showing the location of Tomé‐Açu municipality (in red) (C) satellite imagery (Google Earth) of AGF19 and AGF27 and the native forest within Tomé‐Açu municipality in spatial regards to Belém, the capital city of Pará.

### 2.2 Soil Sampling and Physicochemical Analysis

Soil and aboveground litter samples from AGF19, AGF27, and the native forest were collected and transported to the Cellular and Molecular Biology Laboratory at the Center for Nuclear Energy in Agriculture (CENA) at the University of São Paulo (USP). Sampling was performed at five points in each agroforestry area, with a central point and four additional points in each cardinal direction, 25 m from the central point. Sampling points avoided the agroforest border by at least 20 m. Due to the structure and location of the native forest, only three points were sampled 50 m apart from each other in a straight line, avoiding buffer and transition zones. Soil was sampled at three depths using a Dutch auger cleaned with 70% alcohol after each sampling point. The depths were labelled as surface (0–10 cm), intermediate (10–20 cm), and deep layer (20–30 cm). Small trenches (30 × 30 × 30 cm) were excavated in each site for soil collection in each depth. Soil cores were collected using volumetric rings (5 × 5 cm) with an Uhland‐type auger for soil physical characterization. For chemical, textural and molecular analysis, one sample for each depth was collected in plastic bags, totalizing 15 biological replicates per agroforestry system and 9 replicates in the native forest. Volumetric rings were wrapped in plastic film to facilitate transport and preserve soil structure. Additionally, ~5 g of soil from each replicate were portioned in cryotubes, frozen immediately with liquid nitrogen, and stored first in dry ice and later at −80°C for posterior DNA extraction. Aboveground leaf litter was sampled using a PVC square frame (25 × 25 cm) on each spot where the trenches for soil sampling were dug, totalizing five replicates per agroforestry and three in the forest area. Wood pieces and branches were manually removed and there was no distinction between leaves from different tree species during sampling. Litter deposition was slightly denser in the native forest area, with greater observed diversity of leaves' morphologies. Paper bags were used to store the collected litter material, later dried at 50°C for 48 h before shipping to analysis of chemical composition. These samples (soil and litter) were then shipped to Agrilab (Brazil), where physical and chemical parameters were assessed according to Almeida (2008) and Raij et al. (2001), respectively. For litter analysis, 250 g of dried material from each sample were used to assess the chemical profile of the plant tissues.

### 2.3 Soil DNA Extraction, Amplicon Sequencing and Bioinformatics

Samples for molecular analysis were collected on site using cryotubes tubes (Kasvi, Brazil). The tubes were instantly frozen in liquid nitrogen after sampling. At the Cellular and Molecular Biology Laboratory, whole genomic DNA was extracted from each sample. The extraction was carried out with the DNeasy PowerLyzer PowerSoil Kit (Qiagen, Hilden, Germany). DNA quality and concentration were measured using a NanoDrop 2000c spectrophotometer (Thermo Fisher Scientific, Waltham, MA, United States). The V4 region of the 16S rRNA was amplified to assess the abundance of prokaryotes (Bacteria and Archaea) from samples by using the primers 515F and 806R (Caporaso et al. 2011; Apprill et al. 2015; Parada et al. 2016). For assessing fungal community, primers ITS1F and ITS2R were used (White et al. 1990). The paired‐end sequencing with 2 × 300 bp reads was performed in the Illumina NextSeq2000 platform in NGS Genomic Solutions (Brazil).

Raw reads from sequencing were uploaded into the R environment (R Core Team 2025) and processed following the DADA2 pipeline using standard parameters (Callahan et al. 2016). The 16S sequences were organized in amplicon sequence variants (ASVs) and annotated using the SILVA v.138 database (Quast et al. 2013), whereas ITS sequences were annotated using the UNITE database (Abarenkov et al. 2024). Datasets were merged with non‐sequencing data to build an R6 object for use in the microeco package (Liu et al. 2021).

### 2.4 Data Analysis

For soil data, a Shapiro–Wilk normality test indicated that litter chemical data did not follow a normal distribution. Therefore, a Mann–Whitney *U* test was used to assess statistical significance between the different areas. For physicochemical soil parameters, a two‐way repeated‐measures analysis of variance (ANOVA) was used to evaluate the effects of sampling area, soil depth, and their interaction.

For amplicon sequencing data, observed alpha diversity was calculated using the microbiome R package. Differences in observed richness were assessed using a two‐way ANOVA, considering sampling area, soil depth, and their interaction as fixed factors. When significant effects were detected, pairwise comparisons were performed using Tukey's honestly significant difference (HSD) test based on estimated marginal means.

Beta diversity was evaluated separately for prokaryotic and fungal communities using centered log‐ratio (CLR)‐transformed amplicon sequence variant (ASV) abundances and Euclidean distances. Community dissimilarities were visualized by principal coordinates analysis (PCoA), and differences among sampling areas and soil depths were tested by permutational multivariate analysis of variance (PERMANOVA) with 999 permutations using the vegan R package (Anderson 2001; Oksanen et al. 2024). Differential taxonomic abundance among sampling areas was assessed using two complementary approaches implemented in the microeco R package (Liu et al. 2021). Random Forest analysis was used to identify the taxa with the highest discriminatory power among sampling areas, whereas MetaStat was used to detect taxa showing significant differences in relative abundance. Redundancy analysis (RDA) was performed using the vegan package to evaluate the relationships between microbial community composition and soil physicochemical variables (Oksanen et al. 2024).

Potential ecological functions of fungal communities were inferred using FUNGuild v1.0 (Nguyen et al. 2016), whereas potential functional profiles of prokaryotic communities were predicted using FAPROTAX (Sansupa et al. 2021). Differences in the relative abundance of predicted functional groups among sampling areas were evaluated using Kruskal–Wallis tests, and functions with *p* < 0.05 were considered significantly different.

All statistical analyses were performed in R version 4.5.0 (R Core Team 2025). Data manipulation and visualization were conducted using the packages phyloseq (McMurdie and Holmes 2013), microbiome (Lahti and Shetty 2017), microeco (Liu et al. 2021), vegan (Oksanen et al. 2024), rstatix, and ggplot2 (Wickham 2016). Statistical significance was considered at *p* < 0.05 unless otherwise stated.

## 3 Results

### 3.1 Soil and Litter Characteristics

The chemical composition of the soil differed between sites and depths (Table S1). AGF27 showed no detectable concentration of aluminium (Al) in any depth, with statistically higher concentrations of copper (Cu) and pH. AGF19 presented Al in all depths sampled, but lower concentrations than what was determined at the forest area. Many other elements (such as Fe, P and H) showed a decreasing trend with depth, especially on samples from AGF19 and the forest. Concentrations of many parameters varied as if AGF19 represented a transient state between AGF27 and the native forest. AGF27 showed similar concentrations of nutrients between soil depths, with a significant reduction associated with depth mainly for Zn, Mg and Mn. Noteworthy, total organic carbon (TOC) and organic matter (OM) values decreased with depth in all sampled areas.

Soil physical parameters showed minimal variation between all three areas, with the native forest showing slightly less density in all depths (Figure S1). Overall, soil density increased with depth while soil porosity decreased. The litter chemical composition revealed that the highest concentration of nutrients was calcium, followed by nitrogen, potassium, magnesium, and sulphur (Figure S2). AGF27 showed statistically significant differences for higher concentrations of Ca and Cu, while AGF19 showed the highest concentration of B, and the native forest area had the highest concentration of Fe and the lowest of K and Zn.

### 3.2 Microbial Abundance Across Sites

Amplicon sequencing assigned 27,893 bacterial and archaeal amplicon sequence variants (ASV) and 25,927 fungal ASVs. The overall distribution of bacterial phyla was consistent across individual replicates in all sampled areas (Figure 2). Both agroforestry systems differed considerably from the native forest area. The main groups found and the fluctuation trends per depth were similar within AGF19 and AGF27, with surface replicates showing greater similarity per site. Archaea was more abundant in the agroforestry systems, but overall represented less than 1% of the relative abundance assessed in all samples, being represented within ‘Others’. The most abundant classified bacterial phyla were Proteobacteria in all sampled areas, with the native forest possessing a considerably high percentage of unclassified abundance (from 45% to ~60%). Actinobacteria, Acidobacteria, Firmicutes, Planctomycetes, and Chloroflexi were the remaining leading phyla in the agroforestry soils, accounting together for over 75% of the overall abundance in all depths. One sample from AGF27 at the deepest soil evaluated shown a contrasting pattern with a dominance of ~87% of Proteobacteria. In the forest area, besides Proteobacteria, only Acidobacteria had a relative abundance of over 3%. On some samples of both AGF19 and AGF27, Firmicutes (particularly at 10–20 cm), and Chloroflexi (especially at 20–30 cm) increased with depth, while Actinobacteria showed a slight reduction on the deepest samples. Proteobacteria remained as the predominant group in all sites and depths sampled.

**FIGURE 2:**
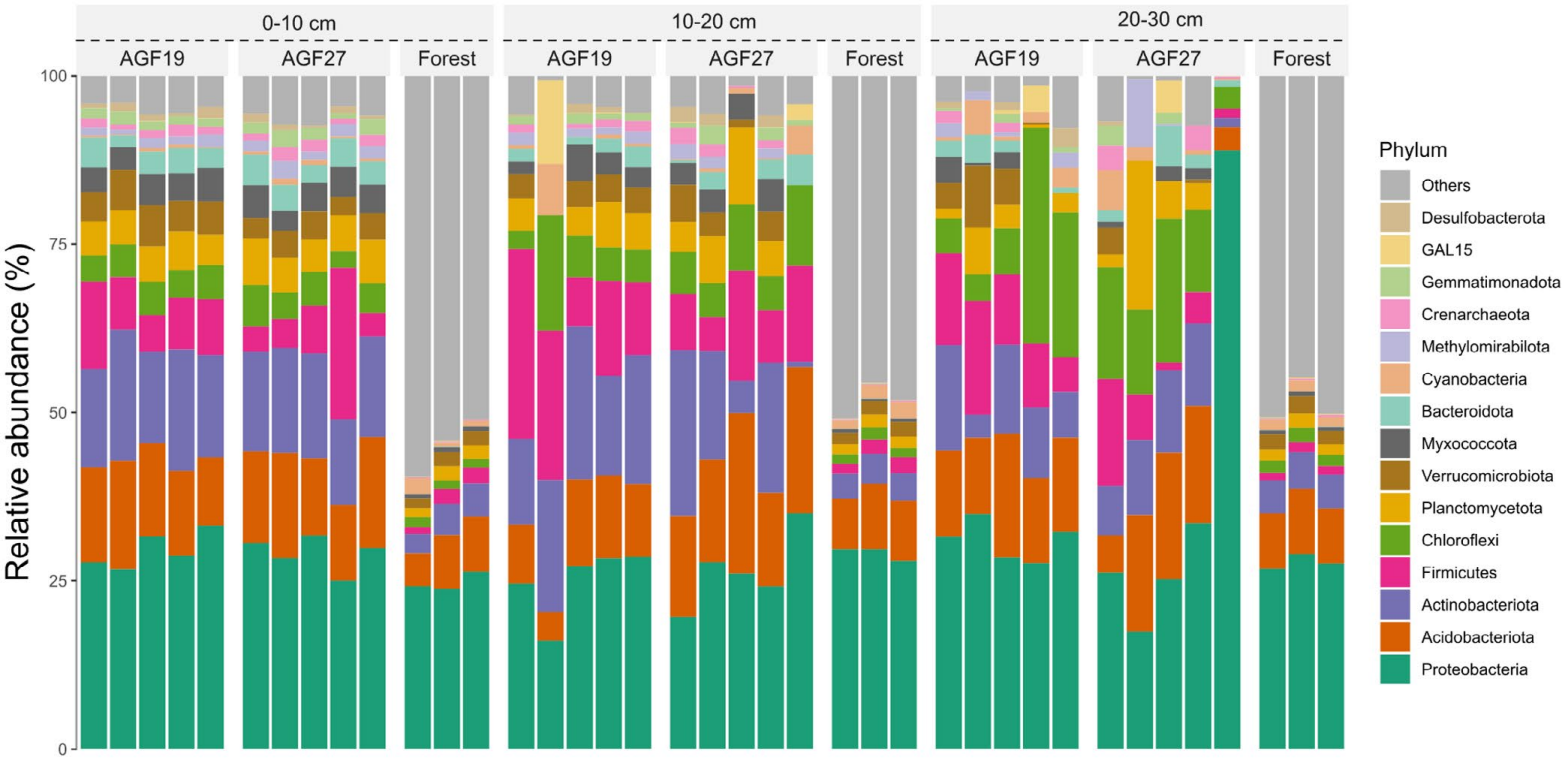
Relative abundance (%) from domains Archaea and Bacteria (shown by phylum) on each replicate of AGF19, AGF27 and Native Forest area per soil depth sampled. Each phylum is represented by a different colour within the descriptive caption provided on the right. The top 15 phyla with the highest relative abundance are individually represented, with the remaining data (less abundant phylum or unclassified) being condensed in Others.

At the genus level, AGF19 and AGF27 were more similar to each other than the forest (Figure S3). The majority of the most abundant genera (*Acidibacter*, *Candidatus Solibacter*, *Candidatus Koribacter*, *Candidatus Xiphinematobacter*, *Bryobacter*, *Conexibacter* and *Mycobacterium*) were more frequent in the forest, with no significant difference when comparing AGF19 and AGF27. Conversely, *Gaiella* and *Bradyrhizobium* were most abundant in the agroforestry systems. *Acidothermus* was found to be more abundant in the forest, followed by AGF19 and AGF27.

The fungal phyla distribution was consistent across AGF19 and AGF27, diverging from the forest fungi community (Figure 3). On all sampled sites, the intra‐treatment fungal composition was similar, seemingly influenced by depth. For instance, Ascomycota, with a proportion of over 60% of the total ASVs retrieved on the surface (0–10 cm) samples, falls to less than 50% at 20–30 cm soil depth for most replicates in the agroforestry systems. Oppositely, in the Forest, Basidiomycota represents the most abundant fungal phylum, particularly in the 0–20 cm samples. The dominance in relative abundance at the fungal community for both agroforestry replicates was replaced by Basidiomycota as the predominant phylum in a depth gradient, varying from less than 10% at the surface to more than 50% in most replicates in the 20–30 cm soil samples. Once again, the forest showed a contrary trend, with Basidiomycota dominance in the surface being slightly replaced by Ascomycota at the deepest sample. Overall, the fungal communities on AGF19 and AGF27 at the deepest layer evaluated (20–30 cm) were less diverse (reduced portion of phylum being represented in the overall total relative abundance), compared to the top layers of the soil.

**FIGURE 3:**
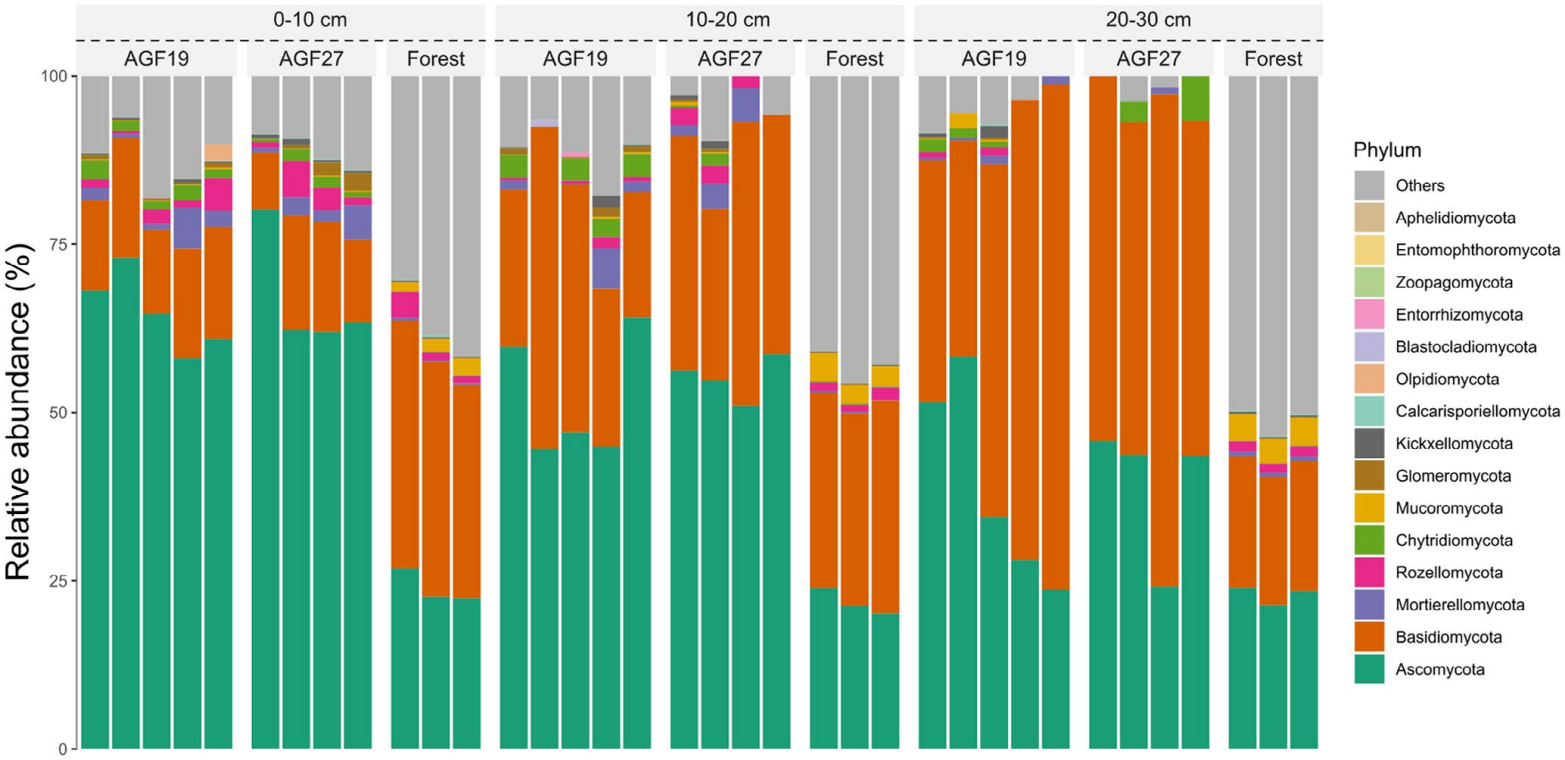
Relative abundance (%) at Phylum level of Fungi on each replicate of AGF19, AGF27 and Native Forest area per soil depth sampled. Each phylum is represented by a different colour within the descriptive caption provided on the right. The top 15 phyla with the highest relative abundance are individually represented, with the remaining data (less abundant phylum or unclassified) being condensed in Others. It was not possible to extract data from the ITS sequencing for one of the sampling points in the AGF27 site, represented only by the remaining four sampling points. One AGF27 replicate did not yield usable fungal sequencing data and was therefore excluded from the analysis. Consequently, four sampling points are shown for AGF27.

In the genus level, one of the most abundant taxonomically placed taxa was *Penicillium*, occurring in similar relative abundances in both AGF19 and AGF27 (Figure S4). The native forest area showed a higher abundance of several genera, including *Clitocybula*, *Rectipilus*, *Genonema*, *Trechispora*, *Mortierella* and *Entoloma*. A similarity between the fungal community in AGF19 and the forest was observed, with *Apiotrichum, Mariannaea* and *Pseudallescheria* occurring in a higher abundance in these areas when compared to AGF27.

### 3.3 Diversity Indexes and Microbial Composition Correlated With Soil Physicochemical Traits

The observed alpha diversity of bacteria and fungi varied across soil depths and land‐use systems (Figure S5). Bacterial diversity was highest in the surface soil (0–10 cm) and decreased with depth mainly in AGF19 and the native forest. No significant differences were detected among AGF19, AGF27, and the forest at 0–20 cm. In the deepest samples, AGF19 varied significantly only when compared to AGF27 (Table S6). Fungal diversity was statistically highest in the forest area at all sampled depths (Table S7). In AGF19 and AGF27, there was a decline in observed alpha diversity with depth.

Beta diversity revealed that the forest has microbial communities that are compositionally distinct within depth and from those found in the agroforestry systems (Figure 4A,B and Table 1). Among the two agroforestry systems, the bacterial community structure was more strongly influenced by area × depth (Table 1), with a trend of per depth clustering that blended both AFS. For fungi, area, depth and their relationship differed statistically (Table 1), with the depths of each AFS more strongly clustered together, representing a greater similarity within each agroforestry.

**FIGURE 4:**
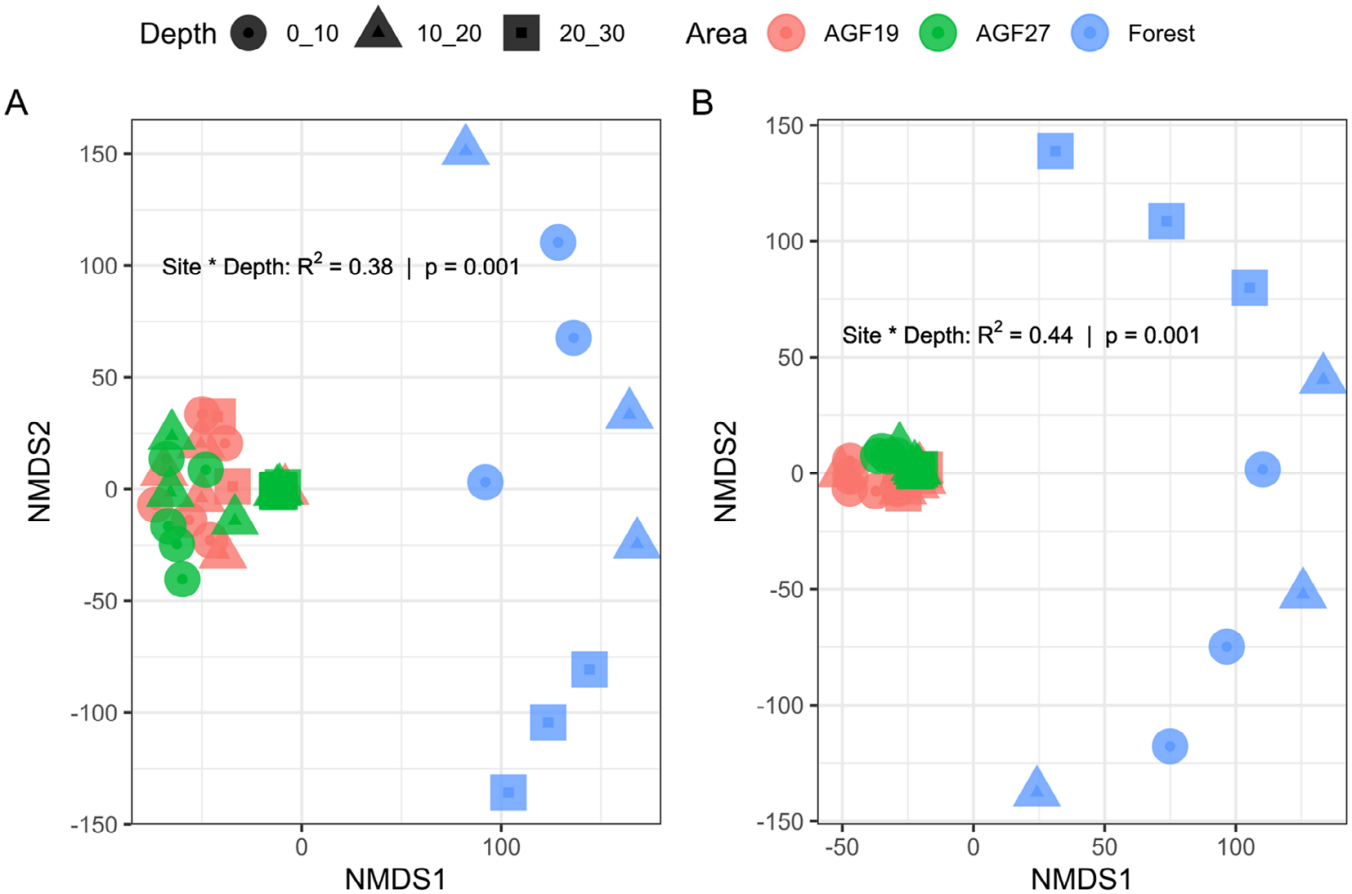
Overall comparisons of microbial communities based on principal coordinates analysis (PCoA), depicting clusters of bacterial (A) and fungi (B) communities in samples from 19‐year‐old agroforestry (red), 27‐year‐old agroforestry (green), and Native Forest (blue) groups. Each point represents a microbial community sampled. Points closer to each other represent similar microbial communities, while points farther from each other represent dissimilar microbial communities. *R* ^2^ correlation between site and depth was 0.38 for bacterial data and 0.44 for fungal data.

**TABLE 1:** PERMANOVA of bacterial and fungal community composition as a function of study area and soil depth.

Dataset	Source	df	Sum of squares	*R* ^2^	Pseudo‐*F*	*p*
Bacteria	Area	2	187,905	0.214	5.180	**0.001**
	Depth	2	47,070	0.054	1.298	0.067
	Area × depth	4	97,336	0.111	1.342	**0.027**
	Residual	30	544,138	0.621	—	—
	Total	38	876,448	1.000	—	—
Fungi	Area	2	169,858	0.246	5.939	**0.001**
	Depth	2	47,341	0.069	1.655	**0.015**
	Area × Depth	4	87,835	0.127	1.536	**0.011**
	Residual	27	386,088	0.559	—	—
	Total	35	691,122	1.000	—	—

*Note:* Permutational multivariate analysis of variance (PERMANOVA) evaluating the effects of study area, soil depth, and their interaction on bacterial and fungal community composition. Analyses were performed on Euclidean distance matrices calculated from centered log‐ratio (CLR)‐transformed ASV abundance data using the adonis2() function in the vegan package with 999 permutations. Results correspond to sequential (Type I) sums of squares. Statistically significant effects (*p* < 0.05) are shown in bold.

Placing bacteria (Figure 5) and fungi (Figure 6) along with chemical factors, the RDA revealed distinct microbial compositions associated with different soil depths and land‐use systems. *Gaiella* (bacteria), *Talaromyces*, *Hansfordia*, *Lepiota*, and *Lipomyces* (fungi) showed strong associations with specific soil properties, such as pH, Ca, P, K, Zn, Cu, Mn and Mg within all depths of AGF19 and AGF27. On the other hand, higher levels of B, S, H, and Al were drivers for the separation of the Forest group, with a dissimilar distribution of most bacterial taxon and a few fungi, such as *Trichoderma*, *Clitocybula*, *Rectipilus* and *Genonema*.

**FIGURE 5:**
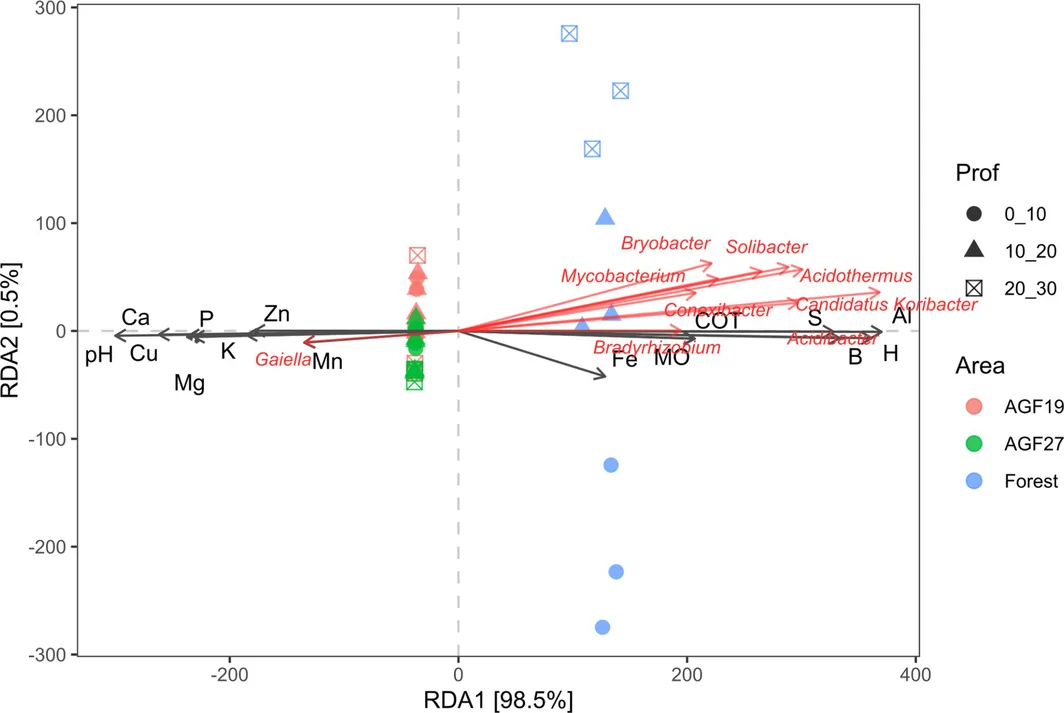
Redundancy Analysis (RDA) biplot showing the relationships between bacterial composition, soil physicochemical properties, and land‐use systems (AGF19, AGF27, and Forest) across three soil depths (0–10 cm, 10–20 cm, and 20–30 cm). Symbols represent samples, coloured by land‐use system: AGF19 (green), AGF27 (orange), and Forest (purple), while different shapes indicate soil depths. Biotic and abiotic factors are represented in arrows, which direction relates to positive correlation between factors and samples.

**FIGURE 6:**
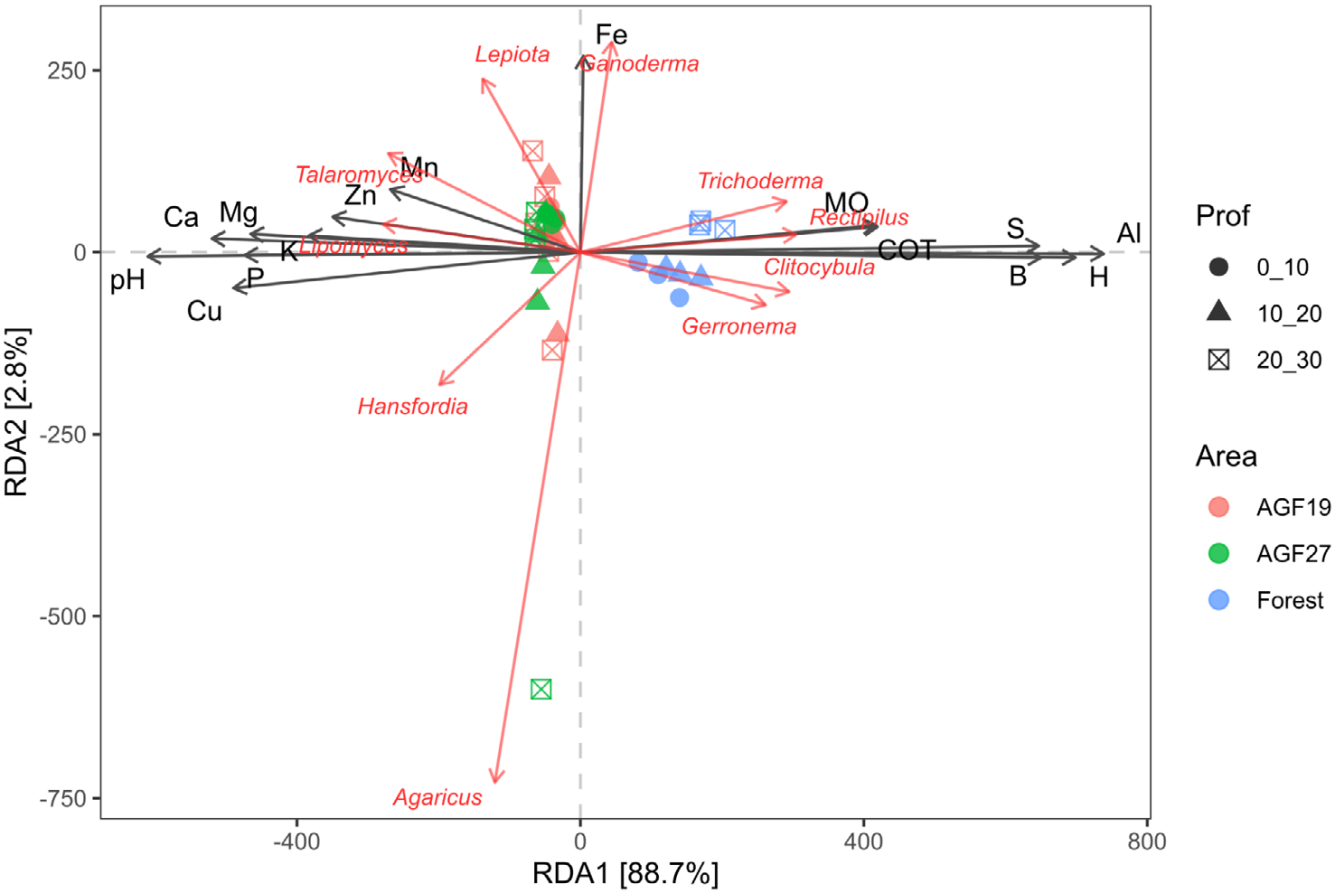
Redundancy Analysis (RDA) biplot showing the relationships between fungal composition, soil physicochemical properties, and land‐use systems (AGF19, AGF27, and Forest) across three soil depths (0–10 cm, 10–20 cm, and 20–30 cm). Symbols represent samples, coloured by land‐use system: AGF19 (green), AGF27 (orange), and Forest (purple), while different shapes indicate soil depths. Biotic and abiotic factors are represented in arrows, which direction relates to positive correlation between factors and samples.

### 3.4 Differential Abundance and Potential Microbial Functions

Many bacterial genera (*Acidothermus*, *Bryobacter*, *Mycobacterium*, *Candidatus Solibacter*, *Candidatus Koribacter*, *Candidatus Xiphinematobacter* and *Conexibacter*) were identified as key biomarkers for the forest area (Figure S6). In AGF19, *Bacillus* and *Gaiella* were highlighted as important indicator genera, whereas AGF27 had a Proteobacteria taxon identified as MND1 and *Arenimonas* as main biomarkers. The random forest analysis described several important fungal biomarkers (Figure S7) for AGF19 (such as *Pyrenochaetopsis*, *Ascodesmis* and *Curvularia*) and the native forest (like *Apiotrichum*, *Tricoderma*, *Mariannaea* and *Pseudallescheria*). In contrast, AGF27 was only linked to *Fusarium*, a potentially phytopathogenic genus within the Ascomycota phylum.

As for bacterial functional groups (Figure 7), FAPROTAX highlighted some statistically significant differences between the three sites studied in regard to the predicted metabolic functional potential associated with the microbial diversity assessed by amplicon sequencing (Table S8). Some statistically significant trends worth noting are the decrease of nitrogen fixation with depth in all areas, the overall higher abundance of nitrate reduction taxa in both AFS when compared to the forest and the greater abundance in all depths (considering absolute values) of iron respiration and intracellular parasites at all depths in the forest site.

**FIGURE 7:**
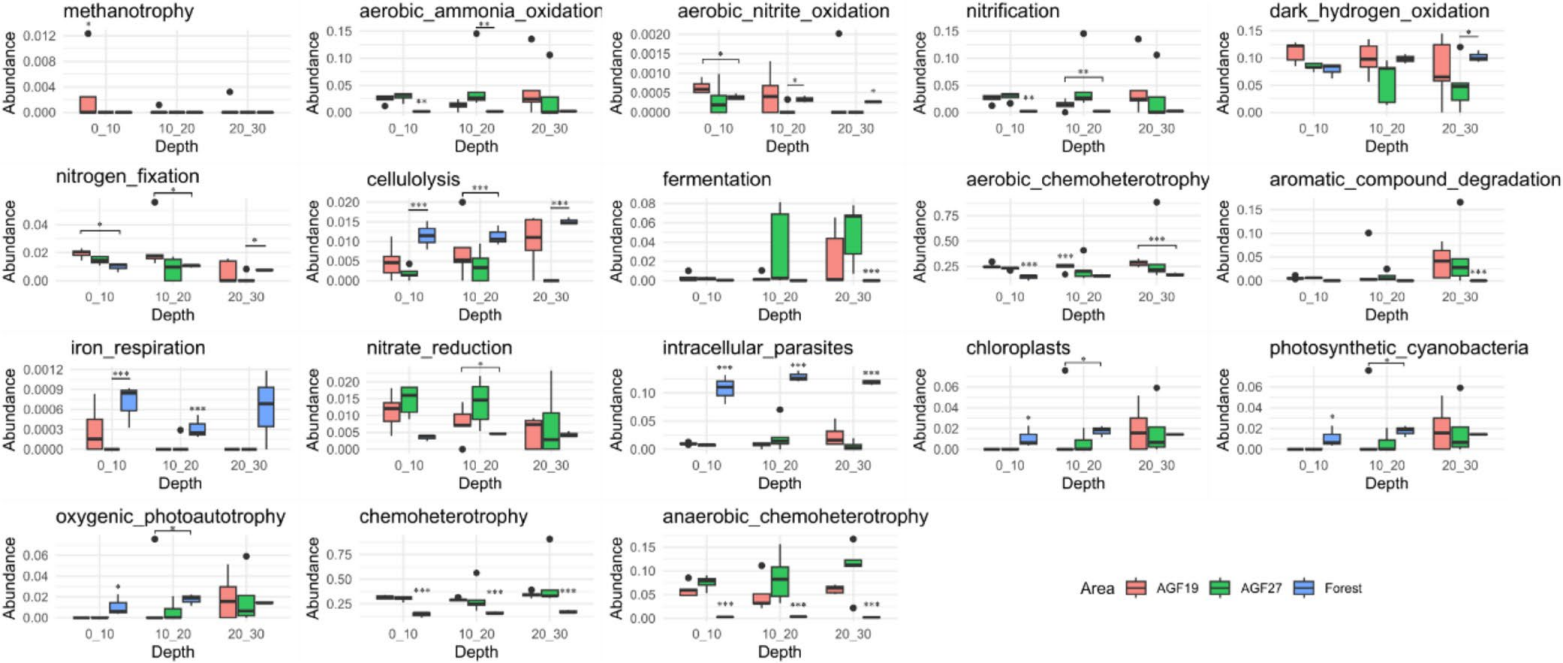
Functional annotation of prokaryotic taxa (FAPROTAX) analysis evaluating the abundance of microbiota in AGF19, AGF27 and the native forest reference area per depth within different metabolic functions, colour coded per area. Seventeen distinct functions are presented, with an independent *Y*‐axis scale for each function based on the observed data distribution, including outliers, to facilitate visualization of differences in abundance across functions. Significance levels are denoted as follows: *p* < 0.05 (*), *p* < 0.01 (**), and *p* < 0.001 (***).

Fungal guild assignment (Figure 8) based on FUNGuild revealed notable potential trends, including a significantly lower abundance of pathotrophs in the native forest (Table S9). In contrast, plant pathogenic fungi were statistically more abundant in both agroforestry systems, particularly in AGF27 at the deepest soil layer. AGF19 and AGF27 also displayed an overall significantly higher abundance of symbiotrophs, endophytes, soil, wood and plant saprotrophs, whereas leaf saprotrophs were more significantly abundant at the surface and intermediate layers of the native forest. It is important to stress that these data reveal possible trends based on ITS sequencing rather than experimentally demonstrated functions.

**FIGURE 8:**
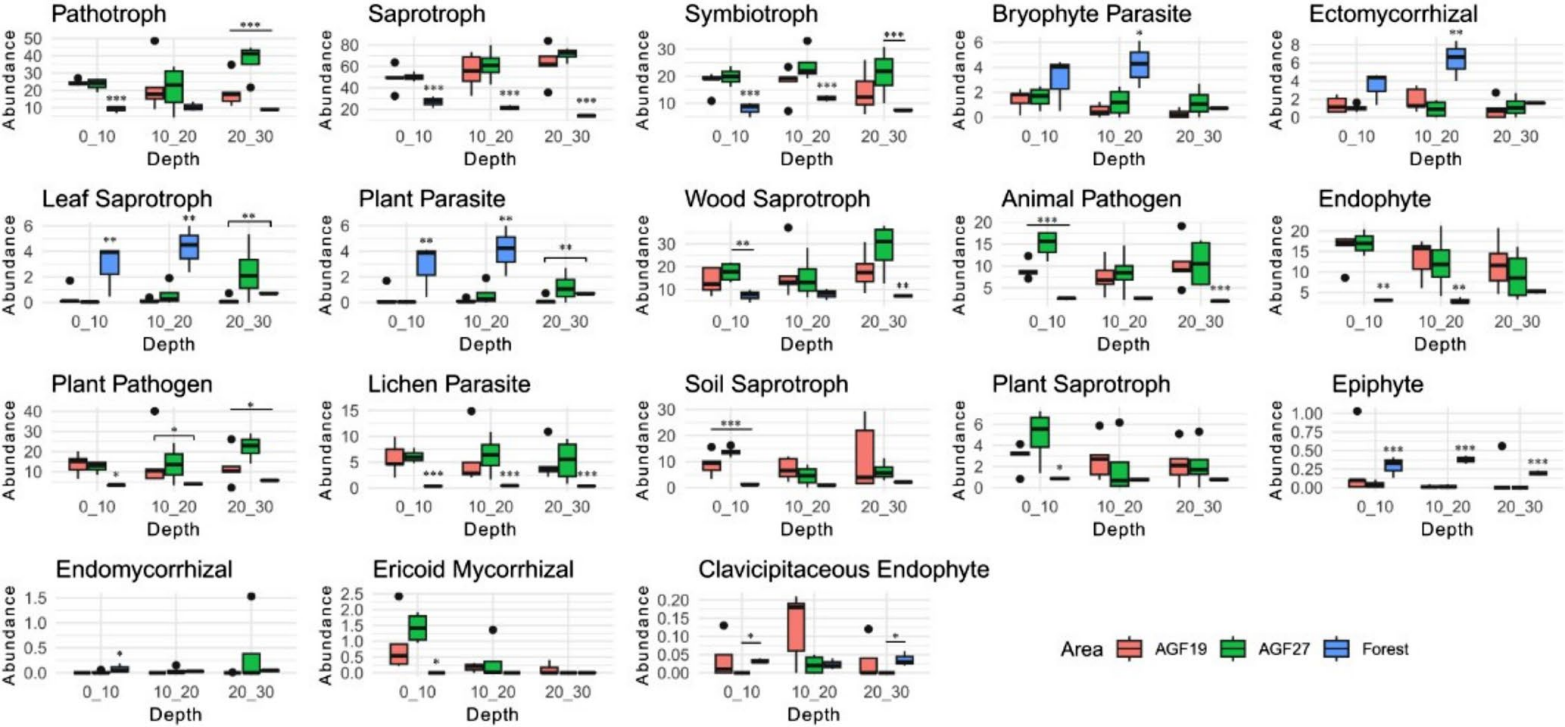
Functional annotation of fungal guilds (FUNGuild) analysis evaluating the abundance of fungi in AGF19, AGF27 and the native forest reference area per depth within different trophic roles, colour coded per area. Eighteen distinct functions are presented, with an independent *Y*‐axis scale for each function based on the observed data distribution, including outliers, to facilitate visualization of differences in abundance across functions. Significance levels are denoted as follows: *p* < 0.05 (*), *p* < 0.01 (**), and *p* < 0.001 (***).

## 4 Discussion

The enrichment of soil organic carbon promotes plant development while contributing to climate change mitigation through carbon sequestration (Celentano et al. 2020). Surface soil (0–10 cm) carbon concentrations in the studied systems (15.1 ± 1.6 g/dm^3^ in the native forest, 12.9 ± 4.2 g/dm^3^ in AGF19, and 10.7 ± 2.6 g/dm^3^ in AGF27; Table S1) were comparable to those reported for other agroforestry systems (AFS) in Tomé‐Açu and the Eastern Amazon (de Carvalho et al. 2014; Celentano et al. 2020), and remained substantially higher than values typically observed in grasslands or bare soils (Li, Liu, Feng, et al. 2022). Similar carbon levels between AGF19 and AGF27 likely reflect the continuous maintenance of litter cover, a defining characteristic of agroforestry systems that enhances nutrient recycling and contributes to soil organic carbon accumulation through decomposition (Osei et al. 2018). Differences in nutrient availability, particularly in AGF27, likely reflect contrasting fertilization practices rather than intrinsic soil characteristics. Potassium and phosphorus concentrations exceeded those previously reported for agroforestry systems in the region (Sousa et al. 2024), whereas native forest values were more comparable to those described for unmanaged soils. Likewise, soil pH in both AFS was higher than in the native forest, consistent with the application of lime to alleviate the naturally acidic conditions of Amazonian soils (Luizão et al. 2007a). Although liming can alter microbial community composition (Yin et al. 2021; Li, Liu, Yao, et al. 2022), its effects on fungal communities remain inconsistent across studies (Kennedy et al. 2005). Lower concentrations of H^+^ and Al^3+^ throughout the soil profile further support previous or ongoing pH correction, particularly in AGF27, highlighting the importance of regular soil monitoring to maintain favourable conditions for crop development while minimizing potential disturbances to the soil microbiome.

Agroforestry systems also improve soil quality by increasing organic matter, enhancing aggregate stability, and reducing erosion (Fahad et al. 2022). Organic matter concentrations in both AFS and the native forest exceeded those previously reported for nearby agroforestry systems, monocultures, and forest soils (Sousa et al. 2024), suggesting that local environmental conditions and management practices can potentially influence soil properties. Similarly, the observed decrease in porosity and increase in bulk density with depth followed patterns previously described for Amazonian agroforestry systems (de Oliveira et al. 2022). The limited use of mechanization likely contributes to preserving soil structure relative to intensive agriculture (Cherubin et al. 2019; de Oliveira et al. 2022), although it also increases labour demands, emphasizing the need for sustainable management practices that balance productivity with soil conservation. Litter cover is another key component of agroforestry systems, sustaining soil moisture and nutrient cycling (Santos et al. 2021). Litter from AGF19 and AGF27 contained higher concentrations of K, Ca and Zn than the native forest, whereas iron was approximately fourfold higher in forest litter, consistent with previous reports for Amazonian soils (Luizão et al. 2007b). Because litter was analysed as a composite pool rather than by plant species, the contribution of vegetation composition to its chemical profile could not be evaluated. Nevertheless, in highly diverse tropical forests, the chemical characteristics of the litter stock may be more relevant to soil fertility than species identity alone (Rabelo et al. 2024). Previous studies demonstrating efficient nutrient recycling in mature cacao‐based agroforestry systems (Pérez‐Flores et al. 2018) further support the importance of maintaining litter cover to sustain nutrient availability and long‐term soil fertility.

As plant development and fruit quality are strongly influenced by soil properties, microbial communities are essential for sustaining productive agroecosystems (Todeschini et al. 2018). The high microbial diversity observed in both agroforestry systems (AFS) and the native forest indicates well‐preserved soil microbiome conditions (Saleem et al. 2019), although most of the recovered ASVs could only be assigned to higher taxonomic ranks, highlighting the need for continued microbial isolation and taxonomic characterization (Spain et al. 2009; Dextro et al. 2021). Microbial diversity patterns varied between soil depths and microbial groups. Although fungal diversity tended to be higher in the native forest, bacterial diversity was generally higher in agroforestry soils, particularly at AGF27. These differences may reflect contrasting ecological filters acting on fungal and bacterial communities (Schmidt et al. 2019), as fungi are often strongly associated with plant diversity, organic matter inputs, and mycorrhizal interactions, whereas bacterial communities may respond more rapidly to changes in nutrient availability and soil chemical conditions that can be influenced by agricultural management practices. Likewise, beta diversity clearly separated both AFS from the native forest, consistent with previous studies showing that agroforestry systems develop microbial communities distinct from adjacent natural ecosystems (Banerjee et al. 2016; Liu et al. 2019). Greater values of measured α or β diversities do not necessarily indicate improved functionality or a gradient of healthier communities. Agroforests' soil microbiome should be evaluated on their capacity to support ecosystem functions instead of being expected to replicate unmanaged forest‐like conditions. Literature observations from other AFS seem to strengthen the idea that agroforestry system age influences bacterial community composition (Liu et al. 2019), moving it towards a greater abundance of oligotrophic taxa. However, the age gap between the study‐case sites sampled in this study might not have been enough to cause clear and obvious distinction in the bacterial phylum composition of both agroforests. Still, age alone is unlikely to explain variation patterns, as management practices and land‐use history are also major drivers of microbial succession (Leite et al. 2023). Collectively, our findings suggest that although agroforestry promotes diverse and resilient microbial communities, these systems establish unique microbial assemblages shaped by both current management and legacy effects rather than converging towards native forest communities within a few decades (Udawatta et al. 2019; Leite et al. 2023).

Archaea represented less than 1% of the microbial community across all sites, a pattern previously reported for other agroforestry systems and potentially associated with lower greenhouse gas emissions than conventional croplands (Shi et al. 2022; Arndt et al. 2026). Proteobacteria was the most abundant bacterial phylum in both AFS and the native forest area, which differed from the agroforests by showing a co‐dominance between Proteobacteria and Acidobacteria throughout the soil depth profile. Although phosphorus enrichment has been associated with increased Proteobacteria and reduced oligotrophic taxa (Cheng et al. 2020), the forest site had less than 10% of the total phosphorus estimated in both AFSs and still presented a Proteobacteria dominance in its bacterial community, suggesting that nutrient availability alone does not determine community composition. Instead, the contrasting bacterial assemblages observed between agroforestry and forest soils likely result from the combined effects of fertilization, pruning, weeding, and other management practices, emphasizing that intensive management may shift agroforestry microbiomes towards communities more typical of conventional agricultural systems. Microbial diversity generally declined with soil depth, particularly for bacterial communities in AGF19 and fungal communities in both AFS, a pattern consistent with previous studies in croplands, intercropping systems, and other agroforestry systems (Hao et al. 2021; Guo et al. 2021; Wang et al. 2022). Nevertheless, both agroforestry sites maintained a high abundance of predicted bacterial functional groups and fungal guilds relative to the native forest, indicating the preservation of microbial groups potentially associated with key ecosystem processes. Similar improvements in nitrogen cycling, nutrient conservation, and symbiotic microorganisms have been reported following the conversion of croplands to agroforestry (Beule and Karlovsky 2021; Elrys et al. 2023). Together, these findings suggest that management practices may alter microbial community composition without necessarily compromising the ecological functions required to support soil fertility and plant productivity.

At the genus level, bacterial communities were mostly correlated with soil nutrients and pH. Biomarkers identified in AGF19 included *Gaiella*, linked to organic matter turnover (Wei et al. 2024), and *Bacillus*, a genus widely recognized for antimicrobial activity and phosphate solubilization (Saeid et al. 2018; Sousa et al. 2024). *Arenimonas*, a metabolically versatile genus adapted to variable environmental conditions (Shan et al. 2023), characterized AGF27, whereas the native forest was enriched in genera such as *Bryobacter*, *Mycobacterium*, *Candidatus Solibacter*, *Candidatus Koribacter*, and *Acidothermus*, which were associated with organic matter, and other soil chemical parameters. Several of these taxa have been linked with important soil functions in tropical soils, such as organic matter decomposition (Walsh et al. 2019; Wiryawan et al. 2022; Bera et al. 2023). *Bradyrhizobium*, an important nitrogen‐fixing genus commonly associated with legumes (Jaramillo et al. 2013), was abundant in both agroforestry systems and the native forest. Overall, the bacterial communities identified in both AFS are consistent with soils possessing the potential to sustain nutrient cycling and other ecosystem processes. However, because microbial functions were inferred from taxonomic composition rather than directly measured, these results should be interpreted as functional potential rather than evidence of realized ecosystem activity. Fungal communities in this study showed a clear stratification pattern associated with soil depth. Ascomycota dominated the surface layers of both agroforestry systems (AFS), similarly to tropical agroforests in China, where this phylum represented approximately 60% of the fungal community at the surface and decreased slightly in deeper soils (Guo et al. 2019; Zhang et al. 2019). This pattern may be related to the functional role of Ascomycota in organic matter decomposition, including the degradation of plant residues through lignin‐degrading enzymes (Lundell et al. 2010). Conversely, the native forest showed a distinct pattern, with Basidiomycota dominating surface soils and Ascomycota increasing at deeper layers. The Ascomycota/Basidiomycota ratio has been proposed as a qualitative indicator of soil organic carbon dynamics and land‐use effects (Manici et al. 2024). Although AFS did not fully resemble the fungal profile of the native forest, both systems maintained diverse mycobiomes containing potential functional groups involved in decomposition, nutrient cycling, plant interactions and biological regulation. Since fungal taxa respond perceptively to environmental changes, they can serve as potential bioindicators of soil conditions and ecosystem functioning (Warnasuriya et al. 2023).

Fungal genera associated with soil chemical properties further highlighted differences among studied sites. Both AFS harboured taxa with recognized plant growth‐promoting functions, including *Talaromyces*, which produces phytohormones such as indole‐3‐acetic acid (IAA) and contributes to nutrient solubilization (Zapata et al. 2024), and *Hansfordia*, which acts as a mycoparasite and potential biocontrol agent (Maeda et al. 2025). AGF19 presented several fungal biomarkers, including *Ascodesmis, Pseudopithomyces* and *Pyrenochaetopsis* (Figure S7). The increased abundance of *Ascodesmis* may be associated with manure‐based fertilization, as coprophilous fungi are commonly linked to animal‐derived organic inputs (Nguyen et al. 2018; Ibuot et al. 2019). Moreover, the presence of *Pseudopithomyces* and *Pyrenochaetopsis*, both reported as antagonists of phytopathogenic fungi (Surono et al. 2023; García‐Latorre et al. 2024), point to a fungal community associated with soil suppressiveness. Conversely, AGF27 had a higher overall abundance of fungal plant pathogens per depth and presented only *Fusarium* as a biomarker (Figure S7). Many strains of this genus are recognized as threats to several plant species and to food security, as *Fusarium*‐related diseases are difficult to control and cause underdevelopment, yellowing and premature decay (Ekwomadu and Mwanza 2023). Biofertilizers and manure have been linked to soil suppressiveness towards *Fusarium* (Xiong et al. 2017; Yang et al. 2022), highlighting how the differences in management practices can cause a direct impact on soil's microbiome. Lime application and mineral fertilization have been reported to alter fungal communities, including increasing Ascomycota abundance in agricultural soils (Yin et al. 2021). However, the influence of liming and phosphorus fertilisation on fungal community structure remains context‐dependent and varies according to soil characteristics, location and management history (Kennedy et al. 2005; Xia et al. 2020; Li, Liu, Yao, et al. 2022). Therefore, because this study compared individual case‐study sites rather than replicated management treatments, these interpretations should be considered associations rather than direct causal effects.

The native forest exhibited higher abundances of several fungal genera, including *Apiotrichum*, *Clitocybula*, *Gerronema*, *Mortierella*, *Rectipilus* and *Trichoderma*, which were correlated with organic matter, total organic carbon and other soil properties. These taxa include rhizosphere‐associated, mycorrhizal and saprotrophic fungi involved in nutrient cycling and plant development (Šnajdr et al. 2010; Zhao and Zhao 2021). Particularly, *Mortierella*, *Trichoderma* and *Apiotrichum* have been associated with improved nutrient acquisition, phosphorus mobilization, pathogen suppression and production of specialized metabolites (Boughalleb‐M'Hamdi et al. 2018; Ozimek and Hanaka 2020; Xu et al. 2025). Overall, the differences observed between AFS and native forest fungal communities likely represent the combined effects of management practices, environmental conditions and unmeasured site‐specific factors. Thus, the main conclusion is that, at the sampling moment, the studied agroforestry soils supported distinct fungal assemblages compared with the native forest, while maintaining diverse microbial communities regularly associated with relevant ecological functions. Agroforestry systems are increasingly recognized as important strategies for achieving several United Nations Sustainable Development Goals (SDGs), particularly those related to food security, responsible production, climate mitigation and biodiversity conservation (Goparaju et al. 2020). Likewise, soil microbial communities are emerging as fundamental components of sustainable agriculture and ecosystem resilience (Crowther et al. 2024). By integrating agroforestry and microbiological approaches, studies such as this contribute to understanding how agricultural systems can be designed to promote soil biological functioning while supporting sustainable food production and environmental conservation.

Working in real‐life settings, particularly within a private property, poses specific challenges that a greenhouse experiment does not face. Both farms sampled are part of a cooperative effort in the Amazon called Tomé‐Açu Mixed Agricultural Cooperative (CAMTA), which has existed since 1929. These farmers have promoted traditional knowledge by testing plant combinations and management regimes over the last 50 years, with extremely positive results (CAMTA 2024). When comparing management methods, it would be extremely interesting to be able to describe the exact frequency and dosages of fertilizers and lime applied per hectare in both AFS, better describing each management type. However, this information was deemed private by landowners, who were already kind enough to allow us the possibility of sampling their farms. The incomplete management records and absence of replicated agroforestry sites limit our capacity to reach more robust conclusions regarding direct management effects on soil properties. Therefore, our data provide a time‐point glimpse of soil physicochemical and microbial conditions in two neighbouring agroforests with distinct management in the Eastern Amazon region, showing that they differ from a native forest reference site.

## 5 Conclusion

This study provides observational inferences that agree with literature evidence that agroforestry management can potentially influence soil physicochemical properties and microbial communities. Despite an 8‐year age difference, AGF19 demonstrated equivalent soil fertility and lower abundance of potentially pathogenic fungi compared to AGF27, which uses chemical inputs and more frequent liming. It is plausible to explain part of these differences by considering that organic‐oriented management may not only reduce operational costs (associated with the acquisition of chemical fertilizers) but also favour soil abiotic and biotic indicators towards a distinct condition when compared to conventional management regimes. Neither management strategy led to a forest‐like soil condition. The contrasts found between both AFS sampled and the reference native forest could be associated with specific approaches, such as external nutrient input and soil acidity control, that distance agroforestry from natural habitats in order to support agricultural practices. By integrating periodic soil evaluations that include microbiological assessment into land management, producers and policymakers can unlock the full sustainability potential of agroforestry as a climate‐resilient and ecologically balanced alternative to conventional systems. Future efforts should prioritise the use of microbial bioindicators in soil monitoring across a large range of well‐described management practices, promoting actions that align economic viability with environmental conservancy in Amazonian agroforestry systems and elsewhere across the globe.

## Author Contributions

**Rafael Barty Dextro:** conceptualization, investigation, formal analysis, visualization, writing – original draft, writing – review and editing, methodology, data curation. **Maike Rossmann:** methodology, investigation, writing – review and editing. **Anderson Santos Freitas:** formal analysis, visualization, writing – review and editing, software. **Juan Andres Domini:** investigation, writing – review and editing. **Guilherme Lucio Martins:** writing – review and editing. **Uchida Shoichiro:** formal analysis, visualization. **Luiz Lehmann Coutinho:** validation, resources, writing – review and editing. **André Freire Cruz:** funding acquisition, writing – review and editing. **Naoaki Ono:** writing – review and editing, formal analysis, software, visualization, supervision. **Siu Mui Tsai:** conceptualization, funding acquisition, writing – review and editing, supervision, project administration.

## Funding

This work was supported by Fundação de Amparo à Pesquisa do Estado de São Paulo, 2022/13186‐4, 2023/08974‐6, 2024/03870‐0, 2021/10626‐0, 2024/06334‐2. Japan Science and Technology Agency, JK20231012. Coordenação de Aperfeiçoamento de Pessoal de Nível Superior, Finance Code 001. Conselho Nacional de Desenvolvimento Científico e Tecnológico, 384253/2023‐7, 130852/2023‐6, 314806/2021‐0, 300104/2025‐1, 423437/2021‐6.

## Ethics Statement

All scientific ethical practices and national regulations were respected during the development of this work.

## Conflicts of Interest

The authors declare no conflicts of interest.

## Supporting information


**Figure S1:** Comparison of the physical characteristics (density, microporosity and macroporosity and total porosity) of the three soil depths of each site sampled. No error bars were added to forest since the data represents a single sampling point.
**Figure S2:** Comparison of the chemical composition of the litter material sampled on AGF19, AGF27 and the forest area. P, S, Mg, K, N and Ca = g kg^−1^ soil; Mn, Fe and Zn = mg kg^−1^ soil; Cu and B = mg kg^−1^ soil. The asterisk * represent significant statistical difference based on Mann–Whitney *U* test whereas ns means no significant difference.
**Figure S3:** Genera diversity (Relative Abundance, %) of the top 25 prokaryotes (Bacteria and Archaea), colour coded per area. The asterisk * represent significant statistical difference whereas ns means no significant difference.
**Figure S4:** Genera diversity (Relative Abundance, %) of the top 25 Fungi, colour coded per area. The asterisk * represent significant statistical difference whereas ns means no significant difference.
**Figure S5:** Observed α diversity of Bacteria (left) and Fungi (right) displayed per depth and colour coded per area. (*) Denotes statistically significant difference (*p* < 0.01).
**Figure S6:** Random forests analysis for biomarkers in each area (left panel) Top 20 microbial genera for classifying each area (right panel) Relative abundance (%) of each genus per area. Data are shown in mean ± standard deviation.
**Figure S7:** Random forests analysis for biomarkers in each area (left panel) Top 20 fungal genera for classifying each area (right panel) Relative abundance (%) of each genus per area. Data are shown in mean ± standard deviation.
**Table S1:** Comparison of the chemical parameters on three soil depths of AGF19, AGF27, and the Native Forest area. Different letters represent a significant statistical difference between sites (ANOVA, *p* < 0.05).
**Table S2:** Raw data obtained for soil physical parameters per sampling point (P) and per depth (d1, 0–10 cm; d2, 10–20 cm; d3, 20–30 cm) in both agroforestry systems and in the forest area.
**Table S3:** Raw data obtained for soil chemical minerals (B, Cu, Fe, Mn, P, S and Zn) presented as mg/dm^3^ soil per sampling point (P) and per depth (d1, 0–10 cm; d2, 10–20 cm; d3, 20–30 cm) in both agroforestry systems and in the forest area.
**Table S4:** Raw data obtained for soil chemical parameters (pH, Organic Matter, Total Organic Carbon, Al, Ca, H, K and Mg) per sampling point (P) and per depth (d1, 0‐10 cm; d2, 10–20 cm; d3, 20‐30 cm) in both agroforestry systems and in the forest area.
**Table S5:** Raw data obtained for litter chemical composition per sampling point (P) in both agroforestry systems and in the forest area.
**Table S6:** Tukey‐adjusted pairwise comparisons of observed bacterial richness among areas within each soil depth.
**Table S7:** Tukey‐adjusted pairwise comparisons of observed fungal richness among areas within each soil depth.
**Table S8:** Kruskal–Wallis test results for bacterial functional groups among study areas.
**Table S9:** Kruskal–Wallis test results for FUNGuild‐predicted fungal trophic modes and guilds among the three study areas.

## Data Availability

Raw data concerning litter and soil physicochemical parameters per sampling point are presented in Tables S2–S5. Reads obtained from amplicon sequencing were deposited at the Sequence Read Archive (SRA) of the National Center for Biotechnology Information (NCBI) and can be downloaded within the BioProject PRJNA1267153.
