# Supplementary material for: Management‐Driven Effects in Soil Physicochemical Properties and Microbial Communities in Eastern Amazonian Agroforests
Source: Environ Microbiol Rep. 2026 Oct 11;18(5):e70432. doi: 10.1111/1758-2229.70432 (PMC13654599; doi:10.1111/1758-2229.70432)
Supplement: Supplementary file 1 [file EMI4-18-e70432-s001.docx]

**Management-driven effects in soil physicochemical properties and microbial communities in Eastern Amazonian agroforests**

Rafael Barty Dextro^1*^; Maike Rossmann^1^; Anderson Santos Freitas^1^; Juan Andres Domini^1^; Guilherme Lucio Martins^1,2^; Uchida Shoichiro^3^; Luiz Lehmann Coutinho^2^; André Freire Cruz^4^; Naoaki Ono^3^; Siu Mui Tsai^1^

^1^Centre for Nuclear Energy in Agriculture (CENA), University of São Paulo (USP), Piracicaba, Brazil

^2^Superior School of Agronomy “Luiz de Queiroz” (ESALQ), University of São Paulo (USP), Piracicaba, Brazil

^3^Data Sciences Center, Nara Institute of Science and Technology (NAIST), Nara, Japan

^4^Graduate School of Life and Environmental Sciences, Kyoto Prefectural University (KPU), Kyoto, Japan

*Corresponding author: rbdextro@usp.br

Running title: Potential management effects on agroforests soils

Supplementary Material

Supplementary Figure S1. Comparison of the physical characteristics (density, microporosity and microporosity, and total porosity,) of the three soil depths of each site sampled. No error bars were added to forest since the data represents a single sampling point.

Supplementary Figure S2. Comparison of the chemical composition of the litter material sampled on AGF19, AGF27 and the forest area. P, S, Mg, K, N and Ca = g.kg-1Soil; Mn, Fe and Zn = mg.kg-1Soil; Cu and B = mg.kg-1Soil. The asterisk * represent significant statistical difference based on Mann-Whitney U test whereas ns means no significant difference.

Supplementary Figure S3. Genera diversity (Relative Abundance, %) of the top 25 prokaryotes (Bacteria and Archaea), color coded per area. The asterisk * represent significant statistical difference whereas ns means no significant difference.

Supplementary Figure S4. Genera diversity (Relative Abundance, %) of the top 25 Fungi, color coded per area. The asterisk * represent significant statistical difference whereas ns means no significant difference.

Supplementary Figure S5. Observed α diversity of Bacteria (left) and Fungi (right) displayed per depth and color coded per area. (*) denotes statistically significant difference (p < 0.01)

Supplementary Figure S6. Random forests analysis for biomarkers in each area (left panel) Top 20 microbial genera for classifying each area (right panel) Relative abundance (%) of each genus per area. Data are shown in mean ± standard deviation.

Supplementary Figure S7. Random forests analysis for biomarkers in each area (left panel) Top 20 fungal genera for classifying each area (right panel) Relative abundance (%) of each genus per area. Data are shown in mean ± standard deviation.

| Soil Trait | AGF19 | | | AGF27 | | | Forest | | |
| --- | --- | --- | --- | --- | --- | --- | --- | --- | --- |
|  | **0 – 10 cm** | **10 – 20 cm** | **20 – 30 cm** | **0 – 10 cm** | **10 – 20 cm** | **20 – 30 cm** | **0 – 10 cm** | **10 – 20 cm** | **20 – 30 cm** |
| pH | 4.80 ± 0.39 ^a^ | 4.52 ± 0.56 ^a^ | 4.27 ± 0.52 ^b^ | 5.09 ± 0.03 ^a^ | 5.12 ± 0.03 ^a^ | 5.08 ± 0.03 ^a^ | 3.77 ± 0.09 ^b^ | 3.79 ± 0.11 ^b^ | 3.80 ± 0.10 ^b^ |
| Al (mmolc.dm^3^_Soil_) | 1.13 ± 1.00 ^b^ | 3.13 ± 3.00 ^b^ | 4.13 ± 2.70 ^b^ | 0.0 ± 0.0 | 0.0 ± 0.0 | 0.0 ± 0.0 | 22.00 ± 2.40 ^a^ | 17.80 ± 2.86 ^a^ | 17.20 ± 2.70 ^a^ |
| B (mg.dm^3^_Soil_) | 0.33 ± 0.10 ^b^ | 0.22 ± 0.04 ^b^ | 0.22 ± 0.04 ^b^ | 0.25 ± 0.06 ^b^ | 0.14 ± 0.04 ^b^ | 016 ± 0.04 ^b^ | 0.71 ± 0.09 ^a^ | 0.62 ± 0.14 ^a^ | 0.41 ± 0.14 ^a^ |
| Ca (mmolc.dm^3^_Soil_) | 36.90 ± 23.20 ^a^ | 20.43 ± 15.64 ^a^ | 10.32 ± 7.19 ^ab^ | 36.87 ± 5.64 ^a^ | 26.32 ± 3.87 ^a^ | 24.70 ± 9.08 ^a^ | 1.63 ± 0.83 ^b^ | 1.21 ± 0.69 ^b^ | 1.35 ± 0.79 ^b^ |
| Cu (mg.dm^3^_Soil_) | 0.97 ± 0.48 ^b^ | 0.41 ± 0.21 ^b^ | 0.10 ± 0.07 ^b^ | 1.79 ± 0.27 ^a^ | 2.00 ± 0.56 ^a^ | 1.45 ± 0.65 ^a^ | 0.07 ± 0.01 ^c^ | 0.06 ± 0.01 ^c^ | 0.05 ± 0.0 ^b^ |
| Fe (mg.dm^3^_Soil_) | 204.75 ± 68.63 ^ab^ | 128.10 ± 29.56 ^a^ | 75.00 ± 12.05 ^a^ | 129.75 ± 22.56 ^b^ | 126.60 ± 43.69 ^a^ | 88.65 ± 29.80 ^a^ | 205.30 ± 28.50 ^a^ | 117.30 ± 23.60 ^a^ | 81.50 ± 7.20 ^a^ |
| H (mmolc.dm^3^_Soil_) | 30.62 ± 4.18 ^b^ | 26.50 ± 7.08 ^b^ | 20.19 ± 2.39 ^b^ | 21.10 ± 3.94 ^c^ | 19.20 ± 2.63 ^b^ | 19.58 ± 1.31 ^b^ | 87.80 ± 10.46 ^a^ | 64.60 ± 14.00 ^a^ | 57.80 ± 7.70 ^a^ |
| K (mmolc.dm^3^_Soil_) | 3.24 ± 1.96 ^a^ | 1.66 ± 0.71 ^a^ | 1.02 ± 0.30 ^ab^ | 3.24 ± 2.05 ^a^ | 2.03 ± 0.69 ^a^ | 1.69 ± 0.45 ^a^ | 1.17 ± 0.11 ^b^ | 0.89 ± 0.02 ^b^ | 0.84 ± 0.07 ^b^ |
| Mg (mmolc.dm^3^_Soil_) | 7.73 ± 3.72 ^a^ | 3.89 ± 2.29 ^a^ | 2.55 ± 1.49 ^ab^ | 7.81 ± 1.99 ^a^ | 4.89 ± 0.84 ^a^ | 3.98 ± 0.53 ^a^ | 2.23 ± 0.63 ^b^ | 1.14 ± 0.20 ^b^ | 0.91 ± 0.33 ^b^ |
| Mn (mg.dm^3^_Soil_) | 4.27 ± 3.29 ^a^ | 1.50 ± 1.22 ^a^ | 0.53 ± 0.49 ^a^ | 2.97 ± 1.36 ^a^ | 1.13 ± 0.62 ^a^ | 0.70 ± 0.55 ^a^ | 1.89 ± 0.34 ^a^ | 1.28 ± 0.67 ^a^ | 1.06 ± 0.42 ^a^ |
| P (mg.dm^3^_Soil_) | 120.38 ± 84.40 ^a^ | 54.11 ± 51.38 ^ab^ | 14.84 ± 12.08 ^b^ | 98.15 ± 22.80 ^a^ | 94.99 ± 35.13 ^a^ | 107.27 ± 55.76 ^a^ | 7.40 ± 1.65 ^b^ | 3.50 ± 0.92 ^b^ | 2.40 ± 1.20 ^b^ |
| S (mg.dm^3^_Soil_) | 8.78 ± 6.17 ^ab^ | 6.45 ± 2.54 ^b^ | 7.25 ± 3.44 ^b^ | 5.37 ± 2.01 ^b^ | 3.07 ± 1.27 ^b^ | 2.39 ± 0.74 ^c^ | 21.25 ± 9.42 ^a^ | 19.54 ± 4.06 ^a^ | 16.94 ± 1.77 ^a^ |
| Zn (mg.dm^3^_Soil_) | 4.47 ± 2.25 ^a^ | 1.39 ± 1.07 ^a^ | 0.33 ± 0.15 ^a^ | 4.15 ± 0.70 ^a^ | 1.14 ± 0.63 ^a^ | 0.47 ± 0.19 ^a^ | 0.65 ± 0.23 ^b^ | 0.53 ± 0.08 ^a^ | 0.39 ± 0.08 ^a^ |
| Organic Matter (g.dm^3^_Soil_) | 22.29 ± 7.19 ^ab^ | 10.97 ± 4.52 ^ab^ | 6.60 ± 4.54 ^ab^ | 18.46 ± 4.50 ^b^ | 6.99 ± 1.44 ^b^ | 4.07 ± 1.00 ^b^ | 26.00 ± 2.71 ^a^ | 20.35 ± 5.36 ^a^ | 13.00 ± 2.64 ^a^ |
| Total Organic Carbon (g.dm^3^_Soil_) | 12.96 ± 4.18 ^ab^ | 6.38 ± 2.63 ^ab^ | 3.83 ± 2.64 ^ab^ | 10.73 ± 2.62 ^b^ | 4.06 ± 0.84 ^b^ | 2.37 ± 0.59 ^b^ | 15.13 ± 1.57 ^a^ | 11.83 ± 3.12 ^a^ | 7.57 ± 1.53 ^a^ |

Supplementary Table S1. Comparison of the chemical parameters on three soil depths of AGF19, AGF27, and the Native Forest area. Different letters represent a significant statistical difference between sites (ANOVA, p<0.05).

Supplementary Table S2. Raw data obtained for soil physical parameters per sampling point (P) and per depth (d1, 0-10cm; d2, 10-20cm; d3, 20-30cm) in both agroforestry systems and in the forest area.

| AGF19 | Density  (g cm³) | Microporosity (cm³) | Macro porosity (cm³) | Total porosity (cm³) | AGF27 | Density  (g cm³) | Microporosity (cm³) | Macro porosity (cm³) | Total porosity (cm³) |
| --- | --- | --- | --- | --- | --- | --- | --- | --- | --- |
| P1d1 | 1.09 | 0.47 | 0.09 | 0.56 | *P1d1* | 1.14 | 0.40 | 0.03 | 0.44 |
| P1d2 | 1.63 | 0.30 | 0.04 | 0.34 | *P1d2* | 1.44 | 0.30 | 0.05 | 0.35 |
| P1d3 | 1.51 | 0.29 | 0.05 | 0.33 | *P1d3* | 1.99 | 0.27 | 0.06 | 0.33 |
| P2d1 | 1.14 | 0.42 | 0.06 | 0.48 | *P2d1* | 1.21 | 0.39 | 0.07 | 0.46 |
| P2d2 | 1.48 | 0.27 | 0.03 | 0.31 | *P2d2* | 1.47 | 0.27 | 0.04 | 0.31 |
| P2d3 | 1.55 | 0.28 | 0.03 | 0.31 | *P2d3* | 1.02 | 0.31 | 0.05 | 0.36 |
| P3d1 | 1.31 | 0.38 | 0.05 | 0.43 | *P3d1* | 1.00 | 0.38 | 0.06 | 0.44 |
| P3d2 | 1.56 | 0.26 | 0.06 | 0.31 | *P3d2* | 1.59 | 0.24 | 0.08 | 0.32 |
| P3d3 | 1.53 | 0.28 | 0.05 | 0.33 | *P3d3* | 1.80 | 0.30 | 0.04 | 0.35 |
| P4d1 | 0.95 | 0.40 | 0.07 | 0.47 | *P4d1* | 0.93 | 0.42 | 0.05 | 0.47 |
| P4d2 | 1.24 | 0.35 | 0.03 | 0.38 | *P4d2* | 1.27 | 0.33 | 0.05 | 0.38 |
| P4d3 | 1.48 | 0.27 | 0.03 | 0.30 | *P4d3* | 1.25 | 0.29 | 0.03 | 0.33 |
| P5d1 | 1.20 | 0.39 | 0.06 | 0.45 | *P5d1* | 1.11 | 0.44 | 0.06 | 0.50 |
| P5d2 | 1.40 | 0.32 | 0.03 | 0.35 | *P5d2* | 1.32 | 0.35 | 0.05 | 0.39 |
| P5d3 | 1.47 | 0.29 | 0.03 | 0.32 | *P5d3* | 1.52 | 0.31 | 0.04 | 0.34 |

| Forest | Density  (g cm³) | Microporosity (cm³) | Macro porosity (cm³) | Total porosity (cm³) |
| --- | --- | --- | --- | --- |
| P1d1 | 0.87 | 0.40 | 0.05 | 0.45 |
| P1d2 | 1.10 | 0.36 | 0.11 | 0.47 |
| P1d3 | 1.11 | 0.28 | 0.05 | 0.33 |

Supplementary Table S3. Raw data obtained for soil chemical minerals (B, Cu, Fe, Mn, P, S and Zn) presented as mg/dm³_Soil_ per sampling point (P) and per depth (d1, 0-10cm; d2, 10-20cm; d3, 20-30cm) in both agroforestry systems and in the forest area.

| AGF19 | B | Cu | Fe | Mn | P | S | Zn |  | AGF27 | B | Cu | Fe | Mn | P | S | Zn |
| --- | --- | --- | --- | --- | --- | --- | --- | --- | --- | --- | --- | --- | --- | --- | --- | --- |
| P1d1 | 0.45 | 1.3 | 121 | 7.3 | 226 | 6 | 4.2 |  | P1d1 | 0.35 | 1.7 | 164 | 1.8 | 123 | 4 | 3.8 |
| P1d2 | 0.27 | 0.7 | 92 | 3.3 | 128 | 4 | 2.4 |  | P1d2 | 0.15 | 2.0 | 195 | 1.5 | 132 | 2 | 0.8 |
| P1d3 | 0.23 | 0.2 | 67 | 1.3 | 35 | 3 | 0.5 |  | P1d3 | 0.11 | 1.7 | 138 | 1.5 | 127 | 1 | 0.7 |
| P2d1 | 0.31 | 0.3 | 205 | 2.2 | 50 | 6 | 2.9 |  | P2d1 | 0.24 | 1.4 | 120 | 2.0 | 86 | 4 | 4.0 |
| P2d2 | 0.18 | 0.2 | 157 | 0.7 | 12 | 8 | 0.6 |  | P2d2 | 0.11 | 1.4 | 129 | 0.7 | 48 | 2 | 1.0 |
| P2d3 | 0.16 | 0.1 | 95 | 0.3 | 7 | 9 | 0.4 |  | P2d3 | 0.11 | 0.5 | 69 | 0.2 | 16 | 3 | 0.2 |
| P3d1 | 0.23 | 1.1 | 240 | 1.3 | 125 | 7 | 5.0 |  | P3d1 | 0.23 | 2.1 | 134 | 5.2 | 80 | 9 | 5.0 |
| P3d2 | 0.23 | 0.4 | 160 | 0.5 | 42 | 5 | 1.2 |  | P3d2 | 0.15 | 2.6 | 129 | 2.0 | 69 | 4 | 2.3 |
| P3d3 | 0.21 | 0.1 | 69 | 0.2 | 12 | 7 | 0.3 |  | P3d3 | 0.20 | 2.2 | 84 | 1.0 | 103 | 3 | 0.6 |
| P4d1 | 0.43 | 1.5 | 161 | 8.3 | 177 | 20 | 8.0 |  | P4d1 | 0.21 | 1.9 | 102 | 2.5 | 79 | 4 | 3.3 |
| P4d2 | 0.17 | 0.5 | 115 | 2.2 | 83 | 10 | 2.6 |  | P4d2 | 0.10 | 2.5 | 101 | 1.0 | 118 | 4 | 0.9 |
| P4d3 | 0.25 | 0.1 | 77 | 0.7 | 17 | 6 | 0.3 |  | P4d3 | 0.18 | 1.7 | 63 | 0.3 | 166 | 2 | 0.3 |
| P5d1 | 0.25 | 0.7 | 298 | 2.2 | 24 | 5 | 2.2 |  | P5d1 | 0.22 | 1.9 | 129 | 3.3 | 123 | 6 | 4.7 |
| P5d2 | 0.24 | 0.2 | 117 | 0.8 | 6 | 5 | 0.2 |  | P5d2 | 0.19 | 1.5 | 79 | 0.5 | 108 | 4 | 0.7 |
| P5d3 | 0.26 | 0.1 | 67 | 0.2 | 4 | 12 | 0.1 |  | P5d3 | 0.18 | 1.3 | 90 | 0.5 | 124 | 3 | 0.6 |

| Forest | B | Cu | Fe | Mn | P | S | Zn |
| --- | --- | --- | --- | --- | --- | --- | --- |
| P1d1 | 0.66 | 0.07 | 195 | 2.2 | 7.0 | 31.9 | 0.54 |
| P1d2 | 0.70 | 0.07 | 109 | 1.7 | 3.3 | 24.0 | 0.44 |
| P1d3 | 0.57 | 0.05 | 89 | 1.0 | 3.8 | 18.5 | 0.42 |
| P2d1 | 0.81 | 0.07 | 237 | 2.0 | 9.2 | 14.2 | 0.92 |
| P2d2 | 0.72 | 0.05 | 144 | 0.5 | 4.5 | 16.1 | 0.58 |
| P2d3 | 0.29 | 0.05 | 80 | 0.7 | 1.8 | 15.0 | 0.30 |
| P3d1 | 0.65 | 0.05 | 183 | 1.5 | 5.9 | 17.6 | 0.50 |
| P3d2 | 0.46 | 0.05 | 99 | 1.7 | 2.7 | 18.5 | 0.56 |
| P3d3 | 0.36 | 0.05 | 75 | 1.5 | 1.6 | 17.4 | 0.46 |

Supplementary Table S4. Raw data obtained for soil chemical parameters (pH, Organic Matter, Total Organic Carbon, Al, Ca, H, K and Mg) per sampling point (P) and per depth (d1, 0-10cm; d2, 10-20cm; d3, 20-30cm) in both agroforestry systems and in the forest area.

| AGF19 | Al ³ | Ca | H | K | Mg | pH | O.M. | T.O.C. |  | AGF27 | Al ³ | Ca | H | K | Mg | pH | O.M. | T.O.C. |
| --- | --- | --- | --- | --- | --- | --- | --- | --- | --- | --- | --- | --- | --- | --- | --- | --- | --- | --- |
| P1d1 | 0 | 66 | 32 | 2.32 | 10 | 5.1 | 26 | 15.3 |  | P1d1 | 0 | 36 | 22 | 6.81 | 7 | 5.1 | 18 | 10.3 |
| P1d2 | 0 | 43 | 25 | 1.52 | 7 | 5.1 | 18 | 10.4 |  | P1d2 | 0 | 30 | 21 | 2.88 | 4 | 5.1 | 6 | 3.6 |
| P1d3 | 0 | 23 | 17 | 1.44 | 5 | 5.1 | 15 | 8.5 |  | P1d3 | 0 | 36 | 21 | 2.04 | 3 | 5.1 | 4 | 2.2 |
| P2d1 | 3.75 | 18 | 32 | 2.90 | 5 | 4.4 | 13 | 7.5 |  | P2d1 | 0 | 31 | 17 | 1.79 | 6 | 5.1 | 14 | 7.9 |
| P2d2 | 7.5 | 11 | 39 | 1.64 | 2 | 4.1 | 10 | 5.5 |  | P2d2 | 0 | 20 | 16 | 1.20 | 5 | 5.1 | 6 | 3.7 |
| P2d3 | 5 | 7 | 24 | 0.90 | 2 | 4.0 | 5 | 2.7 |  | P2d3 | 0 | 15 | 19 | 1.14 | 4 | 5.1 | 3 | 1.8 |
| P3d1 | 0 | 29 | 27 | 2.36 | 8 | 5.1 | 16 | 9.5 |  | P3d1 | 0 | 46 | 27 | 3.11 | 11 | 5.2 | 26 | 15.0 |
| P3d2 | 3.125 | 11 | 21 | 1.49 | 3 | 4.4 | 7 | 4.0 |  | P3d2 | 0 | 27 | 23 | 2.28 | 6 | 5.2 | 9 | 5.4 |
| P3d3 | 4.375 | 5 | 21 | 0.91 | 2 | 4.0 | 4 | 2.2 |  | P3d3 | 0 | 25 | 21 | 1.76 | 4 | 5.1 | 4 | 2.5 |
| P4d1 | 0 | 56 | 36 | 6.98 | 12 | 5.1 | 29 | 17.0 |  | P4d1 | 0 | 34 | 19 | 2.13 | 7 | 5.1 | 18 | 10.7 |
| P4d2 | 0 | 30 | 26 | 2.89 | 6 | 5.1 | 13 | 7.5 |  | P4d2 | 0 | 27 | 18 | 1.47 | 5 | 5.1 | 8 | 4.4 |
| P4d3 | 3.75 | 9 | 19 | 1.18 | 3 | 4.4 | 4 | 2.5 |  | P4d3 | 0 | 30 | 18 | 1.33 | 4 | 5.1 | 4 | 2.1 |
| P5d1 | 1.875 | 15 | 26 | 1.62 | 3 | 4.4 | 27 | 15.5 |  | P5d1 | 0 | 37 | 21 | 2.38 | 8 | 5.1 | 17 | 9.7 |
| P5d2 | 5 | 7 | 22 | 0.77 | 1 | 4.0 | 8 | 4.5 |  | P5d2 | 0 | 28 | 18 | 2.34 | 4 | 5.1 | 6 | 3.2 |
| P5d3 | 7.5 | 8 | 20 | 0.67 | 1 | 3.8 | 6 | 3.3 |  | P5d3 | 0 | 17 | 19 | 2.19 | 4 | 5.1 | 6 | 3.3 |

| Forest | Al ³ | Ca | H | K | Mg | pH | O.M. | T.O.C. |
| --- | --- | --- | --- | --- | --- | --- | --- | --- |
| P1d1 | 23.4 | 2.4 | 98 | 1.23 | 2.7 | 3.68 | 23 | 13.7 |
| P1d2 | 20.8 | 1.3 | 80 | 0.91 | 1.4 | 3.71 | 16 | 9.4 |
| P1d3 | 20.3 | 2.0 | 61 | 0.88 | 1.3 | 3.70 | 15 | 8.8 |
| P2d1 | 23.4 | 1.7 | 88 | 1.24 | 2.5 | 3.76 | 29 | 16.8 |
| P2d2 | 17.7 | 1.9 | 55 | 0.90 | 1.1 | 3.73 | 26 | 15.3 |
| P2d3 | 15.6 | 0.5 | 49 | 0.76 | 0.7 | 3.78 | 10 | 5.9 |
| P3d1 | 19.3 | 0.8 | 77 | 1.05 | 1.5 | 3.87 | 25 | 14.8 |
| P3d2 | 15.1 | 0.5 | 57 | 0.88 | 1.0 | 3.92 | 18 | 10.7 |
| P3d3 | 15.6 | 1.5 | 63 | 0.89 | 0.8 | 3.91 | 13 | 8.0 |

Al ³, Ca, H, K and Mg presented as mmolc/dm³_Soil_. Organic matter (O.M.) and Total Organic Carbon (T.O.C.) presented as g/dm³_Soil_.

Supplementary Table S5. Raw data obtained for litter chemical composition per sampling point (P) in both agroforestry systems and in the forest area.

| AGF19 | N | P | K | Ca | Mg | S | Fe | Cu | Zn | Mn | B |
| --- | --- | --- | --- | --- | --- | --- | --- | --- | --- | --- | --- |
| P1 | 12.3 | 0.61 | 4.0 | 18.9 | 4.4 | 2.5 | 276.0 | 10.4 | 112.8 | 259.2 | 38.1 |
| P2 | 12.8 | 0.75 | 2.5 | 14.2 | 2.9 | 2.0 | 468.0 | 12.5 | 891.0 | 273.0 | 40.2 |
| P3 | 13.7 | 0.56 | 3.7 | 18.3 | 6.4 | 1.6 | 508.3 | 8.4 | 708.0 | 309.0 | 31.6 |
| P4 | 13.0 | 0.97 | 9.5 | 22.9 | 6.4 | 2.1 | 436.3 | 7.8 | 726.0 | 82.8 | 52.3 |
| P5 | 11.2 | 0.60 | 8.7 | 10.3 | 3.8 | 1.6 | 530.6 | 7.8 | 75.0 | 262.8 | 45.8 |
| AGF27 |  |  |  |  |  |  |  |  |  |  |  |
| P1 | 11.8 | 1.02 | 5.2 | 22.8 | 4.6 | 1.6 | 366.9 | 16.8 | 288.0 | 259.2 | 23.2 |
| P2 | 12.5 | 0.71 | 4.4 | 24.5 | 4.2 | 1.6 | 469.2 | 13.8 | 324.0 | 300.0 | 27.8 |
| P3 | 13.9 | 0.74 | 3.1 | 25.0 | 5.4 | 2.3 | 493.5 | 15.2 | 312.0 | 375.0 | 26.7 |
| P4 | 12.5 | 0.71 | 2.1 | 23.5 | 5.0 | 2.2 | 637.6 | 18.9 | 300.0 | 384.0 | 29.5 |
| P5 | 13.9 | 1.01 | 3.9 | 22.3 | 4.9 | 2.0 | 299.1 | 13.9 | 1128.0 | 214.0 | 30.4 |
| Forest |  |  |  |  |  |  |  |  |  |  |  |
| P1 | 13.5 | 0.29 | 1.0 | 14.9 | 2.9 | 1.4 | 1464.0 | 8.4 | 17.4 | 747.0 | 30.3 |
| P2 | 14.9 | 0.27 | 1.3 | 10.8 | 2.7 | 2.0 | 2418.0 | 8.4 | 19.8 | 309.0 | 28.8 |
| P3 | 14.3 | 0.26 | 1.0 | 13.1 | 2.2 | 1.7 | 1068.0 | 12.6 | 15.0 | 414.0 | 31.1 |

N, P, K, Ca, Mg, and S presented as g/kg _Litter_. Fe, Cu, Zn, Mn and B presented as mg/kg _Litter_.

Supplementary Table S6. Tukey-adjusted pairwise comparisons of observed bacterial richness among areas within each soil depth.

| **Depth (cm)** | **Comparison** | **Estimate** | **SE** | **t** | **Adjusted *P*** |
| --- | --- | --- | --- | --- | --- |
| **0–10** | AGF19 − AGF27 | −107.2 | 116 | −0.922 | 0.6308 |
|  | AGF19 − Forest | −1.0 | 134 | −0.007 | 1.0000 |
|  | AGF27 − Forest | 106.2 | 134 | 0.791 | 0.7113 |
| **10–20** | AGF19 − AGF27 | −153.8 | 116 | −1.323 | 0.3938 |
|  | AGF19 − Forest | 65.5 | 134 | 0.488 | 0.8777 |
|  | AGF27 − Forest | 219.3 | 134 | 1.633 | 0.2476 |
| **20–30** | AGF19 − AGF27 | −369.6 | 116 | −3.179 | **0.0093** |
|  | AGF19 − Forest | −228.7 | 134 | −1.703 | 0.2205 |
|  | AGF27 − Forest | 140.9 | 134 | 1.050 | 0.5519 |

Pairwise comparisons of estimated marginal means (EMMs) for observed bacterial richness (Observed ASVs) among the three study areas (Forest, AGF19, and AGF27) within each soil depth (0–10, 10–20, and 20–30 cm), based on the fitted two-way ANOVA model (Area × Depth). Estimates represent the difference between the first and second area listed in each comparison. Standard errors (SE), t-ratios, and Tukey-adjusted P-values are reported. Statistically significant differences (P < 0.05) are shown in **bold**.

Supplementary Table S7. Tukey-adjusted pairwise comparisons of observed fungal richness among areas within each soil depth

| **Depth (cm)** | **Comparison** | **Estimate** | **SE** | ***t*** | **Adjusted *P*** |
| --- | --- | --- | --- | --- | --- |
| **0–10** | AGF19 − AGF27 | 36.9 | 137 | 0.268 | 0.9611 |
|  | AGF19 − Forest | −1826.3 | 150 | −12.204 | **<0.0001** |
|  | AGF27 − Forest | −1863.2 | 157 | −11.905 | **<0.0001** |
| **10–20** | AGF19 − AGF27 | 265.1 | 137 | 1.929 | 0.1500 |
|  | AGF19 − Forest | −2218.6 | 150 | −14.825 | **<0.0001** |
|  | AGF27 − Forest | −2483.8 | 157 | −15.870 | **<0.0001** |
| **20–30** | AGF19 − AGF27 | 245.5 | 137 | 1.786 | 0.1933 |
|  | AGF19 − Forest | −2319.7 | 150 | −15.501 | **<0.0001** |
|  | AGF27 − Forest | −2565.2 | 157 | −16.390 | **<0.0001** |

Pairwise comparisons of estimated marginal means (EMMs) for observed fungal richness (Observed ASVs) among the three study areas (Forest, AGF19, and AGF27) within each soil depth (0–10, 10–20, and 20–30 cm), based on the fitted two-way ANOVA model (Area × Depth). Estimates represent the difference between the first and second area listed in each comparison. Standard errors (SE), *t*-ratios, and Tukey-adjusted *P*-values are reported. Statistically significant differences (*P* < 0.05) are shown in **bold**.

Supplementary Table S8. Kruskal–Wallis test results for bacterial functional groups among study areas.

| **Functional group** | **χ²** | **df** | **P-value** | **Significant** |
| --- | --- | --- | --- | --- |
| Methanotrophy | 6.923 | 2 | 0.03138 | Yes (*) |
| Methanogenesis by CO₂ reduction with H₂ | – | – | >0.05 | No |
| Hydrogenotrophic methanogenesis | – | – | >0.05 | No |
| Methanogenesis | – | – | >0.05 | No |
| Methanol oxidation | – | – | >0.05 | No |
| Methylotrophy | – | – | >0.05 | No |
| Aerobic ammonia oxidation | 9.354 | 2 | 0.00931 | Yes (**) |
| Aerobic nitrite oxidation | 8.333 | 2 | 0.01550 | Yes (*) |
| Nitrification | 9.357 | 2 | 0.00929 | Yes (**) |
| Sulfate respiration | – | – | >0.05 | No |
| Respiration of sulfur compounds | – | – | >0.05 | No |
| Nitrate denitrification | – | – | >0.05 | No |
| Nitrite denitrification | – | – | >0.05 | No |
| Denitrification | – | – | >0.05 | No |
| Chitinolysis | – | – | >0.05 | No |
| Dark hydrogen oxidation | 8.797 | 2 | 0.01229 | Yes (*) |
| Nitrogen fixation | 7.733 | 2 | 0.02093 | Yes (*) |
| Nitrite respiration | – | – | >0.05 | No |
| Cellulolysis | 18.390 | 2 | 1.02 × 10⁻⁴ | Yes (***) |
| Dark oxidation of sulfur compounds | – | – | >0.05 | No |
| Fermentation | 16.206 | 2 | 3.03 × 10⁻⁴ | Yes (***) |
| Aerobic chemoheterotrophy | 20.121 | 2 | 4.27 × 10⁻⁵ | Yes (***) |
| Human pathogens (all) | – | – | >0.05 | No |
| Human associated | – | – | >0.05 | No |
| Animal parasites or symbionts | – | – | >0.05 | No |
| Aromatic hydrocarbon degradation | – | – | >0.05 | No |
| Aromatic compound degradation | 18.606 | 2 | 9.12 × 10⁻⁵ | Yes (***) |
| Aliphatic non-methane hydrocarbon degradation | – | – | >0.05 | No |
| Hydrocarbon degradation | – | – | >0.05 | No |
| Iron respiration | 19.121 | 2 | 7.05 × 10⁻⁵ | Yes (***) |
| Nitrate respiration | – | – | >0.05 | No |
| Nitrate reduction | 8.256 | 2 | 0.01612 | Yes (*) |
| Nitrogen respiration | – | – | >0.05 | No |
| Intracellular parasites | 21.839 | 2 | 1.81 × 10⁻⁵ | Yes (***) |
| Predatory or exoparasitic | – | – | >0.05 | No |
| Chloroplasts | 7.329 | 2 | 0.02561 | Yes (*) |
| Nonphotosynthetic cyanobacteria | – | – | >0.05 | No |
| Photosynthetic cyanobacteria | 7.329 | 2 | 0.02561 | Yes (*) |
| Anoxygenic photoautotrophy (S oxidizing) | – | – | >0.05 | No |
| Anoxygenic photoautotrophy | – | – | >0.05 | No |
| Oxygenic photoautotrophy | 7.329 | 2 | 0.02561 | Yes (*) |
| Photoautotrophy | – | – | >0.05 | No |
| Photoheterotrophy | – | – | >0.05 | No |
| Phototrophy | – | – | >0.05 | No |
| Ureolysis | – | – | >0.05 | No |
| Chemoheterotrophy | 20.017 | 2 | 4.50 × 10⁻⁵ | Yes (***) |
| Anaerobic chemoheterotrophy | 23.412 | 2 | 8.25 × 10⁻⁶ | Yes (***) |

Results of Kruskal–Wallis tests comparing the relative abundance of FAPROTAX-predicted bacterial functional groups among the three study areas (Forest, AGF19, and AGF27). Functional groups with P < 0.05 were considered significantly different among areas. For significant functions, the Kruskal–Wallis chi-square statistic (χ²), degrees of freedom (df), and P-value are reported; non-significant functions are indicated as P > 0.05. Significance levels are denoted as follows: P < 0.05 (*), P < 0.01 (**), and P < 0.001 (***).

Supplementary Table S9. Kruskal–Wallis test results for FUNGuild-predicted fungal trophic modes and guilds among the three study areas.

| **Fungal guild** | **χ²** | **df** | **P-value** | **Significant** |
| --- | --- | --- | --- | --- |
| Pathotroph | 16.099 | 2 | 3.192 × 10⁻⁴ | Yes (***) |
| Saprotroph | 20.093 | 2 | 4.333 × 10⁻⁵ | Yes (***) |
| Symbiotroph | 14.977 | 2 | 5.595 × 10⁻⁴ | Yes (***) |
| Bryophyte Parasite | 7.160 | 2 | 0.02788 | Yes (*) |
| Dung Saprotroph | – | – | >0.05 | No |
| Ectomycorrhizal | 10.182 | 2 | 0.00615 | Yes (**) |
| Fungal Parasite | – | – | >0.05 | No |
| Leaf Saprotroph | 12.174 | 2 | 0.00227 | Yes (**) |
| Plant Parasite | 14.214 | 2 | 8.194 × 10⁻⁴ | Yes (**) |
| Wood Saprotroph | 13.649 | 2 | 0.00109 | Yes (**) |
| Animal Pathogen | 18.697 | 2 | 8.709 × 10⁻⁵ | Yes (***) |
| Endophyte | 15.719 | 2 | 3.861 × 10⁻⁴ | Yes (**) |
| Plant Pathogen | 15.325 | 2 | 4.702 × 10⁻⁴ | Yes (*) |
| Lichen Parasite | 17.161 | 2 | 1.877 × 10⁻⁴ | Yes (***) |
| Litter Saprotroph | – | – | >0.05 | No |
| Soil Saprotroph | 14.168 | 2 | 8.384 × 10⁻⁴ | Yes (***) |
| Plant Saprotroph | 8.947 | 2 | 0.01141 | Yes (*) |
| Epiphyte | 15.993 | 2 | 3.366 × 10⁻⁴ | Yes (***) |
| Lichenized | – | – | >0.05 | No |
| Arbuscular Mycorrhizal | – | – | >0.05 | No |
| Endomycorrhizal | 18.331 | 2 | 1.046 × 10⁻⁴ | Yes (*) |
| Ericoid Mycorrhizal | 8.011 | 2 | 0.01822 | Yes (*) |
| Orchid Mycorrhizal | – | – | >0.05 | No |
| Root Associated Biotroph | – | – | >0.05 | No |
| Clavicipitaceous Endophyte | 8.595 | 2 | 0.01360 | Yes (*) |
| Animal Endosymbiont | – | – | >0.05 | No |

Results of Kruskal–Wallis tests comparing the relative abundance of FUNGuild-predicted fungal trophic modes and ecological guilds among the three study areas (Forest, AGF19, and AGF27). Guilds with P < 0.05 were considered significantly different among areas. For significant guilds, the Kruskal–Wallis chi-square statistic (χ²), degrees of freedom (df), and P-value are reported; non-significant guilds are indicated as P > 0.05. Significance levels are denoted as follows: P < 0.05 (*), P < 0.01 (**), and P < 0.001 (***).
